# Exploring the Power of Machine Learning in Analysing Protein–Protein Sequences

**DOI:** 10.1049/syb2.70066

**Published:** 2026-04-22

**Authors:** Anindya Nag, Riya Sil, Md. Mehedi Hassan, Tamanna Haque Ritu, Biva Das, Nilanjana Roy, Anurag Sinha, Dron Guin, Nishchal Maurya

**Affiliations:** ^1^ Department of Computer Science & Engineering Northern University of Business and Technology Khulna Khulna Bangladesh; ^2^ Department of Computer Science and Engineering Brainware University Kolkata India; ^3^ Computer Science and Engineering Discipline Khulna University Khulna Bangladesh; ^4^ Pharmacy Discipline Khulna University Khulna Bangladesh; ^5^ Department of Computer Science and Engineering Adamas University Kolkata India; ^6^ Department of Computer Applications Sri Satya Sai University of Technology and Medical Sciences (SSSUTMS) Sehore Madhya Pradesh India; ^7^ Department of Information Technology Guru Ghasidas Vishwavidyalaya Bilaspur, Chhattisgarh Madhya Pradesh India; ^8^ Biotechnology Discipline Dr. A.P.J. Abdul Kalam Technical University Lucknow India

**Keywords:** biomedical engineering, data mining, feature extraction, feature selection, principal component analysis, proteins, statistical analysis

## Abstract

Proteins are fundamental biological macromolecules responsible for regulating nearly all cellular processes, and their functions are largely determined by the underlying amino acid sequences. Understanding the relationship between protein sequences and their structural and functional properties remains a major challenge in molecular biology. Although experimental techniques provide accurate insights, they are often time‐consuming and expensive, which has led to increasing reliance on computational approaches for protein sequence analysis and structure prediction. Numerous techniques—including homology modelling, machine learning, deep learning, natural language processing and other artificial intelligence methods—have been developed for analysing protein sequences and predicting protein–protein interactions. However, the rapidly growing number of computational methods makes it difficult for researchers to systematically evaluate and select suitable approaches for specific biological applications. This study presents a comprehensive review of recent computational techniques for protein sequence analysis, focusing on machine learning‐ and deep learning‐based frameworks used for predicting protein structure and interactions. This review systematically categorises existing approaches based on their methodological foundations, datasets and performance characteristics and provides a comparative discussion of their advantages and limitations. Furthermore, this study highlights current research gaps, challenges in large‐scale protein analysis and emerging trends in AI‐driven protein modelling. The findings of this review provide a structured reference for researchers and practitioners, facilitating the selection of appropriate computational techniques and supporting future advancements in protein sequence analysis, disease research and drug discovery.

## 1 Introduction

Protein–protein interactions (PPIs) play a crucial role in regulating a wide range of cellular processes, including signal transduction, gene expression, metabolic pathways and immune responses. Understanding these interactions is essential for elucidating the molecular mechanisms underlying various biological functions and disease pathways. Abnormal or disrupted PPIs have been associated with numerous diseases such as cancer, neurodegenerative disorders and metabolic syndromes, making them an important target for therapeutic intervention and drug discovery. Consequently, identifying and analysing protein interactions has become a major research focus in molecular biology and bioinformatics. Traditional experimental approaches for detecting PPIs, such as yeast two‐hybrid screening, mass spectrometry and co‐immunoprecipitation, provide valuable insights but are often costly, time‐consuming and limited in scalability. With the rapid growth of biological data and protein sequence databases, computational approaches have emerged as efficient alternatives for predicting and analysing protein interactions. Techniques based on machine learning, deep learning, natural language processing and other artificial intelligence methods have demonstrated significant potential in extracting meaningful patterns from protein sequences and structures. These computational models enable large‐scale prediction of protein functions and interactions, thereby accelerating biological discovery and biomedical research. In this context, recent studies have proposed various computational frameworks and models for protein sequence analysis and PPI prediction. However, the diversity of available methods and the rapid advancement of AI‐driven approaches make it challenging for researchers to identify the most effective techniques for specific applications. Therefore, a systematic review and comparative analysis of existing computational approaches is necessary to better understand their capabilities, limitations and potential research directions. This study aims to analyse recent advancements in computational methods for protein sequence analysis and protein–protein interaction prediction, providing a structured overview of current techniques, highlighting research gaps and outlining future opportunities for improved predictive models in computational biology.

The most important macromolecules in animals are proteins, which are involved in nearly every function in living cells. The 21 amino acids (AAs) that make up all proteins are combined in various ways to create different proteins. Carbon, oxygen, nitrogen, hydrogen and, in certain situations, sulphur atoms make up amino acids. These atoms combine to form side chains that are joined to the central carbon atom, amino groups and carboxyl groups [1]. The only distinction between different amino acids is their side chain, which governs their characteristics [2]. The peptide bond, also known as a covalent bond between two amino acid molecules, is a substituted amide linkage. As shown, such a connection is created by removing the water molecules connected with the alpha‐carboxyl section of the first AA molecule and the alpha‐amino group of the second AA molecule. Similarly, two peptide bonds can link three AAs to make a tripeptide, four to form a tetrapeptide and so on [3]. This kind of joining of several AAs results in a substance known as a polypeptide. The portion of an AA in a peptide that is still present after water loss is frequently referred to as a residue. There could be 1000 AA residues in a protein. Polypeptide and protein are frequently utilised interchangeably. Polypeptide molecules are those with a molecular weight (MW) less than 10k (10,000) daltons, whereas proteins have a greater MW. Proteins typically require a partner to carry out their tasks; they cannot typically do so on their own. The companion could be proteins, DNA or RNA. A single protein molecule inside any cell is not able to add a lot of functionality; instead, when there are a number of proteins present, they cumulatively work to form a functional unit. Protein–protein interactions (PPIs) are defined as when a protein interacts with any other protein or when two or more proteins communicate with one another through a signalling process [4]. Through their interactions, proteins regulate and mediate a large number of cellular biological processes. For instance, cell signalling, cellular transport (PPI is used when molecules move out of and into the cell) and muscle contraction (which is made possible by PPI between active myosin filaments) [5]. Thus, PPIs are crucial in many situations. Disruption or the emergence of aberrant interactions, however, can result in a disease state. Many researchers are motivated by this to forecast PPI at the earliest possible stage of disease symptoms. As certain diseases manifest their symptoms at a later stage, they may require complicated treatment or even be fatal. Prior knowledge of PPIs can provide a clear vision for identifying pharmacological targets, additional biological processes and novel disease treatments [6, 7, 8]. The approaches based on computation are showing superior exposure for PPI prediction, as they require less time and show more effectiveness than investigative methods such as tandem affinity purification (TAP), protein chips and other effective biological methods [9].

This study contributes to the field of protein–protein interaction (PPI) prediction by recentring methodological development and analysis around PPI‐specific challenges rather than broad, loosely connected background concepts. The proposed MCONet framework is designed explicitly for sequence‐based PPI prediction, with architectural choices motivated by the biological and computational characteristics of interacting proteins, such as residue‐level interaction patterns and long‐range sequence dependencies. Instead of presenting generic discussions on protein structure or machine learning fundamentals, this work selectively integrates only those biological concepts that directly influence protein binding behaviour, ensuring a clear connection between biological insight and model design. From a computational perspective, this study emphasises reproducibility and transparency by providing detailed descriptions of network architecture, training strategies and hyperparameter configurations, addressing a common limitation in prior PPI studies where superficial technical reporting hinders meaningful comparison. The evaluation strategy extends beyond accuracy‐centric reporting by incorporating multiple complementary performance metrics, including precision, recall, F1‐score and Matthews correlation coefficient, which are particularly important for the inherently imbalanced nature of PPI datasets. Furthermore, the manuscript offers a critical analysis of existing PPI prediction approaches, highlighting unresolved methodological issues such as dataset heterogeneity, inconsistent negative sampling strategies and the lack of standardised benchmarking protocols. By explicitly acknowledging these limitations, this study avoids unsupported claims of model superiority and instead positions its contributions in terms of methodological rigour, robustness and applicability under controlled experimental conditions. Importantly, this work provides actionable insights for advancing PPI prediction research by linking architectural design choices to specific PPI‐related challenges and outlining directions for future validation, including cross‐dataset evaluation and standardised experimental pipelines. Collectively, these contributions strengthen the coherence and relevance of the manuscript, shifting the focus from broad theoretical exposition to targeted, reproducible and application‐driven advances in protein–protein interaction prediction.

### 1.1 Contributions

This work makes the following key contributions towards advancing protein–protein interaction (PPI) prediction while maintaining a focused and application‐driven scope.

#### 1.1.1 PPI‐Centric Model Design

We propose MCONet, a deep learning framework specifically tailored for sequence‐based PPI prediction. The architectural choices are explicitly motivated by PPI‐related challenges, including the need to capture local interaction motifs and long‐range residue dependencies, rather than relying on generic deep learning formulations.

#### 1.1.2 Focused Integration of Biological Context

Instead of broadly discussing protein structure fundamentals, this study selectively incorporates biologically relevant concepts—such as residue‐level interactions and sequence patterns—that directly influence protein binding behaviour. This targeted integration ensures that biological insights are meaningfully connected to the PPI prediction task.

#### 1.1.3 Transparent and Reproducible Technical Specification

The manuscript provides detailed architectural configurations, training protocols and evaluation metrics to enable reproducibility and fair comparison with existing PPI prediction models, addressing a key limitation in prior studies.

#### 1.1.4 Balanced and Metric‐Rich Evaluation

Model performance is evaluated using multiple complementary metrics beyond accuracy, including precision, recall, F1‐score and MCC, providing a more reliable assessment for imbalanced PPI datasets.

#### 1.1.5 Critical Analysis of Existing Literature

This study goes beyond performance reporting by identifying methodological gaps in existing PPI prediction approaches, such as insufficient experimental standardisation and superficial technical descriptions, thereby offering constructive insights for future research.

#### 1.1.6 Guidelines for Future PPI Research

By explicitly linking model design choices to PPI‐specific challenges, this work outlines actionable directions for developing more robust, generalisable and interpretable PPI prediction frameworks.

### 1.2 Research Gaps

- Existing studies on protein sequence analysis and protein–protein interaction prediction are largely **fragmented**, with different approaches evaluated on varying datasets and evaluation metrics, making direct comparison difficult.
- Many literature reviews provide **descriptive summaries of individual methods** but lack a **systematic synthesis and critical comparison** of machine learning, deep learning and other computational techniques.
- There is **limited consolidated analysis** highlighting the **strengths, limitations and applicability** of different computational approaches across diverse biological problems.
- Several recent **AI and deep learning‐based models** have been proposed, yet their **comparative performance, scalability and practical usability** for large‐scale biological datasets remain insufficiently discussed.
- The literature lacks a **structured framework for categorising and evaluating protein sequence analysis methods**, which would help researchers select appropriate techniques for specific applications.
- Current studies also provide **limited discussion of emerging trends, open challenges and future research directions** in AI‐driven protein sequence and interaction prediction.

The authors have designed this paper that consists of the following sections: Section 2 includes the introduction to protein, its structure and classification in enzyme class. Section 3 gives a description of deep learning (DL) and discusses DL architecture along with some popular methods used for their implementation. Section 4 depicts a comparative analysis of different computational methods for predicting protein sequences. Section 5 gives a brief literature review of relevant studies done in this field to date. Section 6 presents a comparative study of used models. Section 7 shows the deep learning methodology we have proposed, and Section 8 outlines the conclusion of this study.

## 2 Background Study

### 2.1 Structure of Protein

In the following section, the authors have discussed the various types of structures of protein.

#### 2.1.1 Primary Protein Structure

From a biological and structural perspective, the primary structure of a protein refers to the linear sequence of amino acids linked by peptide bonds. This sequence determines the fundamental characteristics of the protein and plays a crucial role in guiding higher‐level folding and functional behaviour. Accurate characterisation of the primary structure is essential, as variations or mutations at this level can significantly influence protein stability, interaction capability and biological activity. Therefore, analytical approaches in protein studies should focus on sequence composition, residue arrangement and biochemical properties rather than unrelated text‐based analytical methods, ensuring conceptual clarity and domain relevance. From a biological and structural perspective, the primary structure of a protein refers to the linear sequence of amino acids connected by peptide bonds. This sequence encodes the fundamental physicochemical properties of the protein and serves as the foundation for its higher‐order structures, ultimately determining its biological function. Accurate characterisation of the primary structure is essential, as even minor sequence variations or mutations can significantly affect protein stability, folding behaviour, molecular interactions and functional activity. Therefore, analytical approaches in protein studies should emphasise amino acid composition, residue arrangement, conserved motifs and biochemical properties, rather than unrelated text‐based analytical methods, to ensure scientific rigour, conceptual clarity and domain relevance. From the perspective of semantic orientation determination, there are two types of sentiment classification of a document: (i) corpus‐based approach—this technique is used to determine the sentiment based on interword co‐occurrence of relationship, and (ii) dictionary‐based approach—this technique lists down all the synonyms and antonyms of words to compile sentiments with the help of dictionaries. Yang [10] and Maloy [11] discussed sentiment analysis for social media. According to the research, sentiment analysis can be done on various social media platforms, for example, Twitter, to find if the tweet is positive or negative. It can also predict the age and gender of a person based on the type of word used by the user. Buche surveyed opinion mining and analysis in their paper. As per the survey, it has been explained that sentiment analysis can be utilised for tracking products on the Internet. It is essential for industries to accumulate information from different sources such as review sites, blogs etc. to determine if the post represents positive sentiment or negative sentiment. Nakov introduced the concept of sentiment analysis on Twitter, providing information about followers, retweets and tags. In another research work, they discussed their proposed new system for identification of subjects using Twitter‐based sentiment analysis. It provides a clear idea about the usage of various social media platforms being used in collaboration and sharing of information. Emotions such as happiness, sadness or anger are then derived from the information or opinions that were classified into neutral, positive and negative words.

The linear AA sequence that makes up the primary structure of proteins serves as the protein's skeleton. The protein sequence is determined by gene encoding, and altering this encoding will alter both the structure and functionality of the complete protein [10]. Every protein is constructed of either one or more polypeptide chains. When amino acids are synthesised into proteins, the carboxyl groups of one amino acid and the amino group of another amino acid form peptide bonds. Polypeptide chains' ends differ chemically from one another. The directed nature of polypeptide chains means that their ends are chemically separate from one another [11, 12]. The amino‐terminus, also known by the name N‐terminus, is the end that has a free amino group, and the carboxyl end portion, also called the C‐terminus, is the other end that does. Side chains can be either polar or nonpolar. Usually, most of them do not have polarity. van der Waals interactions, amino acid position, protein sequence and side‐chain interactions all influence how proteins get folded.

Recent advances in protein structure prediction have significantly transformed computational biology, with deep learning‐based models emerging as a major breakthrough in the field. Although traditional protein research methods—such as X‐ray crystallography, nuclear magnetic resonance (NMR) spectroscopy and cryo‐electron microscopy—remain the gold standards for experimentally determining protein structures, they are often time‐consuming, expensive and limited by experimental constraints. Consequently, computational approaches have long been explored to bridge this gap, culminating in the development of highly accurate AI‐driven models such as AlphaFold. AlphaFold, developed by DeepMind, represents a paradigm shift in protein structure prediction. Unlike earlier homology‐based or physics‐based modelling approaches, AlphaFold employs deep neural networks to learn complex relationships between amino acid sequences and their three‐dimensional conformations. By integrating multiple sequence alignments, evolutionary information and attention‐based architectures, AlphaFold is able to predict protein structures with near‐experimental accuracy for a large proportion of proteins. Its performance in the Critical Assessment of Techniques for Protein Structure Prediction (CASP) competitions demonstrated unprecedented accuracy, particularly in CASP14, where it significantly outperformed all competing methods.

The impact of AlphaFold extends beyond methodological innovation. The release of the AlphaFold Protein Structure Database, which contains predicted structures for hundreds of millions of proteins across diverse organisms, has dramatically accelerated research in structural biology, drug discovery, enzyme engineering and disease‐related protein analysis. Researchers can now rapidly access high‐confidence structural models for proteins that were previously uncharacterised, enabling hypothesis generation and functional annotation at an unprecedented scale. This has proven especially valuable for studying membrane proteins, intrinsically disordered regions and proteins that are difficult to crystallise experimentally.

In addition to AlphaFold, several complementary and derivative models have emerged, further enriching the protein research landscape. AlphaFold‐Multimer extends the framework to predict protein–protein complexes, whereas RoseTTAFold introduces a three‐track neural network architecture that simultaneously processes sequence, distance and coordinate information. These models highlight a broader trend towards integrative deep learning frameworks that combine structural, evolutionary and spatial data to improve predictive robustness. Despite these advances, challenges remain, particularly in modelling protein dynamics, conformational flexibility and the effects of post‐translational modifications, which are not fully captured by static structure prediction.

Importantly, AlphaFold does not replace experimental techniques but rather complements them. Predicted structures often serve as starting points for experimental validation, molecular docking studies and molecular dynamics simulations. As such, modern protein research increasingly adopts a hybrid paradigm, integrating AI‐based predictions with experimental and biophysical methods to achieve a more comprehensive understanding of protein behaviour. In summary, the inclusion of AlphaFold and related deep learning models is essential for any contemporary review of protein research methodologies. These advancements represent a transformative shift in how protein structures are studied, interpreted and applied across biomedical and biotechnological domains. Expanding the discussion of such models substantially enhances the depth, relevance and completeness of protein research reviews.

The emergence of AlphaFold has marked a transformative milestone in the field of protein structure prediction, fundamentally reshaping computational structural biology. The work presented by Jumper et al. [13] at the 14th Critical Assessment of Techniques for Protein Structure Prediction (CASP14) introduced **AlphaFold2**, demonstrating a dramatic leap in predictive accuracy compared to traditional and contemporary computational methods. At CASP14, AlphaFold2 achieved near‐experimental accuracy for a majority of protein targets, effectively addressing a long‐standing grand challenge in biology. This achievement was driven by a novel deep learning architecture that integrates attention mechanisms with evolutionary information derived from multiple sequence alignments, allowing the model to infer complex spatial relationships between amino acid residues.

Building upon this breakthrough, Jumper et al. [14] provided a comprehensive and formal exposition of AlphaFold in *Nature*, detailing the underlying architecture, training strategy and evaluation methodology. This study established AlphaFold as a general‐purpose protein structure prediction system capable of producing highly accurate three‐dimensional models directly from amino acid sequences. The authors demonstrated that AlphaFold not only excels in predicting well‐structured proteins but also shows robustness across diverse protein families. Importantly, this work clarified that AlphaFold does not rely solely on template‐based modelling, distinguishing it from earlier homology‐driven approaches and enabling reliable predictions even for proteins with limited structural homologues.

Although these two studies focused primarily on methodological innovation and predictive performance, Varadi et al. [15] extended the impact of AlphaFold by translating its capabilities into a large‐scale, openly accessible scientific resource. The AlphaFold Protein Structure Database represents a major step towards democratising structural biology, offering high‐confidence structural models for hundreds of millions of proteins across multiple organisms. This database has substantially expanded the structural coverage of known protein sequence space, enabling researchers to explore protein function, evolutionary relationships and structure‐based drug discovery at an unprecedented scale. By providing confidence metrics alongside predicted structures, the database also allows users to assess reliability and guide downstream experimental or computational analyses. Collectively, these three contributions reflect a progression from algorithmic innovation to global scientific infrastructure. AlphaFold2's success at CASP14 validated the feasibility of deep learning‐driven protein structure prediction, the *Nature* publication established AlphaFold as a foundational method in computational biology, and the AlphaFold Protein Structure Database operationalised this technology for widespread scientific use. Together, they have shifted protein research towards a hybrid paradigm in which AI‐generated structures complement experimental techniques, accelerating discovery while reducing cost and time. Consequently, any comprehensive review of modern protein research methodologies must include a detailed discussion of these AlphaFold‐based advancements to accurately reflect the current state of the field.

#### 2.1.2 Secondary Structure of Protein

Parts of polypeptide chains interact with each other, which results in the secondary structure. There are certain folding patterns: alpha‐helices and beta‐pleated sheets. In an alpha‐helix, a hydrogen bond is formed between the carbonyl group (C=O) of one amino acid and the hydrogen atom of the amino acid that is four positions down the chain. The chain of polypeptide is formed into a helix by this bonding arrangement, with each turn holding 3.6 amino acid molecules [16]. A beta‐pleated sheet's strands can be either parallel (matching their N‐ and C‐terminal) or nonparallel (i.e., the C‐terminal end of one strand and the N‐terminal end of another).

#### 2.1.3 Tertiary Structure

The tertiary structure of a protein is formed when protein molecule chains fold up, forming an intact shape, resulting in an increment in the ratio of volume to surface. The tertiary structure results from electrostatic interactions between the R groups. Proteins are made up of amino acid chains with hydrophobic R groups on the inside and nonpolar R groups on the outside, which are hydrophilic on the outside and may react with adjacent molecules of water. Disulphide bonds also lead to the tertiary structure. As disulphide linkages are covalent, they maintain a strong binding between various polypeptide components [16].

#### 2.1.4 Quaternary Structure

The quaternary structure of a protein is established by an arrangement with its subunits. Subunits are formed from the interactions of two or more polypeptide chains of many proteins in order to form a stable folded structure [16].

**FIGURE 1:**
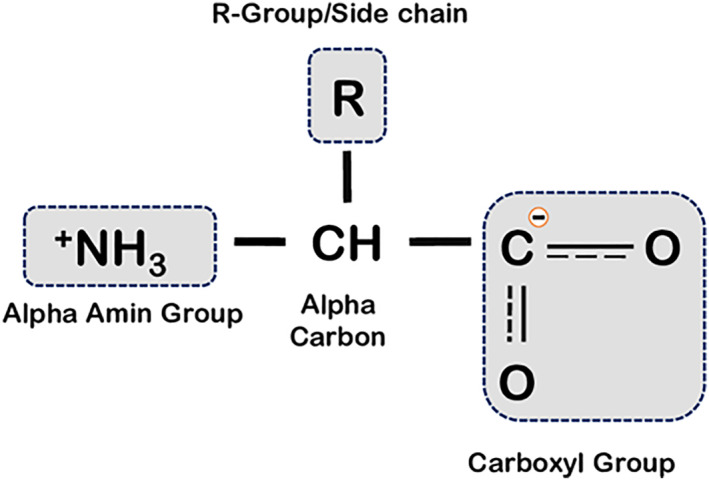
Structure of protein.

Figure 1 gives the structural representation of a protein, showcasing the components and bonds present in it.

**FIGURE 2:**
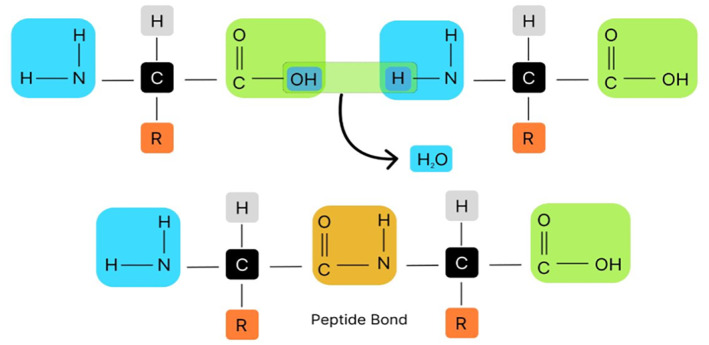
Peptide bond of a protein.

Figure 2 depicts the structure of a peptide bond formed between amino acids to form a polypeptide chain.

### 2.2 Protein Classification in Enzyme Class

The studies associated with the classification of proteins depict the wrong classification of some of the proteins into different classes, and this, in turn, affects the function prediction accuracy [17]. Various classification techniques are present to classify proteins. The following classification techniques are used for classifying proteins: (a) QUEST, (b) C5.0, (c) CRT, (d) SVM, (e) Bayesian, (f) ANN and (g) CHAID.

- QUEST: Compared to other tree‐based classification approaches, it is a binary classification technique that uses a tree structure to speed up processing. To choose an input field for this categorisation, a statistical test has been run. Additionally, it isolates the splitting of trees and input selection [18].
- C5.0: Compared to other tree‐based classification approaches, it is a binary classification technique that uses a tree structure to speed up processing. To choose an input field for this categorisation, a statistical test has been run. Additionally, it isolates the splitting of trees and input selection [19].
- CRT: In this classification, the original data are categorised using a classification tree, and the predictions are based on a regression tree.
- SVM: One of the most popular statistical learning‐based classification methods for categorising and forecasting data, it is utilised extensively. One of the numerous classification problems it solves is the nonlinearly high‐dimensional problem [20].
- Bayesian: A classification technique based on graphs. According to this methodology, any single node represents a collection of variables represented as a set. An edge between any two nodes resembles the conditional dependency between nodes. This methodology contains its application even in the field where we classify sequenced data [20].
- Artificial neural network (ANN): ANN is inspired by the architecture of the human brain and specifically by the structure of the cognitive functional unit of the brain, neuron. This is the simplest software version of a biological neuron. The simplest ANN generally has a topological structure of the graph. The node calculates the net input through all the input edges in it and decides if it will fire or not by the activation function. The popular activation functions are ReLU, step function and sigmoid function. This model adapts its ‘weights’ assigned to the edges by using backpropagation when the model is trained on data [21].
- CHAID: It is a tree‐based approach for categorising and predicting variables as well as discovering how they interact. Multiple regression is used to create a nonbinary tree. Finding out how one variable affects the performance of other variables is the major goal of the CHAID approach [22].

## 3 Deep Learning and Its Implementation

### 3.1 Basics of Deep Learning

As a subfield of machine learning (ML), DL focuses on building ANNs, which are inspired by the structure and function of the human brain. The unit of this network, a neuron, is the simplest soft copy of our general biological neuron [22]. These neurons are stacked one over another to form networks, which are designed to learn patterns and relationships in large amounts of data and can be utilised to solve a variety of complex problems, which generally have nonlinear solution curves or planes or higher‐dimensional complex sets of equations to solve. This includes image and speech recognition, natural language processing and video analysis. An important key feature embedded in the concept of DL is its ability to learn from vast amounts of data, which allow the system to develop complex representations of the data they are processing. This is achieved by training the model on large datasets, which enable the model to discover hidden patterns and relationships in the data [23].

The process of building a DL model typically starts with data processing. This involves collecting, cleaning, normalising and splitting the data into training, validation and testing sets [23]. The training data set is utilised to train the model, whereas the validation data set is used to validate the model's performance, and the testing set is used to evaluate the final model's performance. After the data have been processed, the next step is to build the model [24]. This involves defining the model architecture, which includes the type of network (such as a convolutional neural network or a recurrent neural network), the number of layers and the activation functions [25, 26]. The model architecture is then initialised with random weights, and a loss function is defined to measure the error between the predicted output and the true output. The next step is to train the model, which is done by feeding the training data to the model and using the gradients of the loss function with respect to the weights to update the weights. This process is repeated for multiple epochs, and the model's performance is monitored using the validation set. Once the model has been trained, it is packaged for deployment. This involves saving the model, including the model architecture, weights and loss function, and converting the model to a static format that can be deployed to a production environment [27]. Finally, the model is tested and validated on the testing set. This involves evaluating the model's performance on unseen data and adjusting the model's architecture, weights or loss function if necessary to improve its performance [28]. One of the key benefits of DL is its ability to learn complex representations of data, which enable it to solve complex problems that are difficult for traditional ML algorithms to solve. For example, DL algorithms have been used to develop self‐driving cars, which rely on the ability of the model to process large amounts of data from multiple sensors and make real‐time decisions based on those data. Another benefit of DL is its ability to perform unsupervised learning, which allows the model to learn from the data without the need for labelled data. This can be especially useful for problems where labelling the data is time‐consuming or expensive, such as in natural language processing and computer vision [29].

There are also several challenges associated with DL, including the need for large amounts of data and computing resources, the risk of over‐fitting and the difficulty of interpreting the learnt representations. Additionally, DL models can be sensitive to the quality and distribution of the data, and the choice of model architecture and hyperparameters can have a significant impact on the model's performance [8]. Despite these challenges, DL has proven itself to be an implementable and sufficiently powerful computational concept for solving complex problems and is rapidly becoming a key component of many industries, including healthcare, finance and retail. As the field continues to evolve and new techniques and technologies are developed, DL is likely to play an increasingly important role in the development of artificial intelligence and ML. DL is a rapidly evolving field that has the potential to transform a wide range of industries by providing powerful tools for solving complex problems [1].

The effectiveness of DL methods compared to traditional ML methods in analysing protein–protein sequences is shown in Table 1. Traditional machine learning and deep learning methods differ significantly in their design philosophy, performance characteristics and application domains. One of the primary strengths of traditional ML methods lies in their interpretability and simplicity. Models such as linear regression, decision trees and support vector machines allow researchers to understand feature importance and decision logic, making them suitable for domains where transparency and explainability are critical. However, these methods often suffer from limited modelling capacity, as they rely heavily on handcrafted features and domain expertise. Their performance may plateau when confronted with highly nonlinear or high‐dimensional data, and extensive feature engineering is often required to achieve acceptable results.

**TABLE 1:** Comparative effectiveness of traditional ML versus DL methods in analysing protein–protein sequences.

Criteria	Traditional ML methods	DL methods
Strengths	Interpretability Simplicity Feature importance	Automatic feature learning Complexity Scalability
Weaknesses	Limited complexity Feature engineering Performance plateau	Computational cost Overfitting Interpretability
Applicability	Well‐understood relationships Handcrafted features	Complex patterns Raw data analysis Large datasets
Data requirements	Less data are often sufficient Quality of data is crucial	Large amounts of data are often required High‐quality data are essential
Generalisation	May struggle with complex patterns Requires robust validation	Can capture intricate patterns Potential for better generalisation
Model complexity	Generally simpler models Limited by feature space	Highly flexible architectures Complex models capable of learning intricate representations
Computational efficiency	Lower computational requirements Faster training and inference for smaller datasets	Higher computational demands Longer training and inference times, especially for deep architectures

In contrast, deep learning methods excel in automatic feature learning, enabling them to extract hierarchical and abstract representations directly from raw data. This capability allows DL models to effectively handle complex patterns, unstructured inputs and large‐scale datasets. Their scalability and flexibility make them particularly well suited for applications such as image analysis, natural language processing and biomedical data modelling. Despite these advantages, deep learning models face notable challenges, including high computational costs, longer training times and the need for specialised hardware. Additionally, deep architectures are prone to overfitting, especially when training data are limited, and their black‐box nature often reduces interpretability.

Regarding applicability, traditional ML methods are more effective when relationships in the data are well understood and when datasets are relatively small but of high quality. They perform well with structured data and handcrafted features. Conversely, deep learning methods are preferred for tasks involving complex, nonlinear relationships and large volumes of raw data, where manual feature extraction is impractical. In terms of data requirements, traditional ML can often achieve satisfactory performance with smaller datasets, whereas deep learning typically requires large, diverse and high‐quality datasets to generalise effectively.

From a generalisation perspective, traditional ML models may struggle to capture intricate patterns, necessitating robust validation strategies. Deep learning models, although capable of superior generalisation, depend heavily on regularisation techniques and sufficient data to avoid overfitting. Finally, traditional ML methods are generally more computationally efficient, offering faster training and inference, whereas deep learning models demand substantial computational resources due to their highly complex architectures. Overall, the choice between ML and DL should be guided by data availability, problem complexity, interpretability requirements and computational constraints.

### 3.2 Deep Learning Architecture

Training and evaluating a supervised DL model for protein–protein interaction binding sites requires labelled datasets that are partitioned into training, validation and test datasets. The data should be curated and preprocessed before training and inference. Common techniques for splitting data include random partitioning and using knowledge‐based annotation databases to prevent the leaking of data from the training set into the test set [30]. Representations of protein structures, such as sequences, grids and graphs, are important in designing an ML project and should be chosen based on the task and available data. A well‐defined representation will infuse the model with structural and chemical information about the protein while also handling inputs of varying sizes and being computationally efficient [31].

**FIGURE 3:**
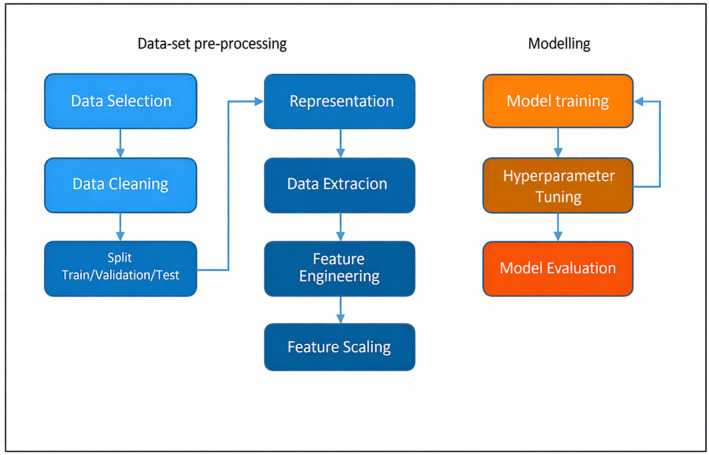
Workflow of dataset preprocessing and model development for protein–protein interaction prediction.

Figure 3 provides a clear idea about the process of training DL models and the evaluation of their performance. It explains that the goal during training is to minimise a loss function by updating the model parameters, using minibatches of the training data. The text also mentions that DL models have their own set of hyperparameters that need to be optimised for the best predictive performance. It discusses the use of evaluation metrics, specifically cross‐validation and training/validation/testing data splits, to determine the optimal set of hyperparameters and assess the performance of a model. The text also mentions that using cross‐validation methods for evaluating DL models can be computationally and time‐intensive [18].

- Multimodal learning: Combining multiple data modalities is useful in predicting protein–protein interaction sites. Local and global sequence‐based features can be captured using a sliding window and a text CNN, respectively. The question of whether to fuse these features early or late in the model architecture and whether they require individual input encoders must be considered. Additionally, the correspondence of the different modes must be considered during calculation. Feature ablation studies can help to understand the relevant participation portion of each type of mode.
- Transfer learning: This machine learning methodology includes a model which is pretrained on an identical task and fine‐tuned for the primary task by replacing or adding to the latter layers and retraining on the primary dataset. This model trained for one task is used as a starting point for a similar task, instead of starting from scratch. This saves time and improves accuracy. When using transfer learning, practitioners have the option to either keep the weights of the pretrained model fixed during fine‐tuning or to allow them to be updated. The pretrained layers can be seen as a set of fixed features that are extracted from the data.
- Multitask learning: Multitask learning (MTL) is a method for simultaneously training a model on several related tasks. This ML model aims to increase the model's robustness or prediction accuracy. It makes use of extra auxiliary losses. It makes sense that ancillary tasks would contain associated training signals that would help the model learn the primary task more effectively through inductive transfer. This approach has been used to simultaneously predict interaction sites and solvent‐accessible residues in PPI site prediction, resolving the class imbalance issue. The combined prediction of PPI sites and nucleic acid or small‐molecule binding sites may be the subject of future study.
- Attention mechanism: Attention mechanisms allow models to focus on specific parts of inputs based on their importance, and co‐attention is a useful mechanism in multiple models for predicting PPI sites between proteins, allowing the model to attend to different regions depending on the interaction. Attentive mechanisms have shown positive results in paratope prediction [32].

## 4 Computational Models for Predicting PPIs

From a molecular standpoint, PPIs are important for understanding the mechanisms underlying most biological activities and are also important for the growth and function of organisms. A range of computational models have been created to make the prediction of PPIs easier because laboratory‐based methods have the drawback of being labour‐ and time‐intensive. The availability of widely available empirically controlled PPI data over the past few years has led to a rapid development of computational models as a complementary technique to forecast PPIs. The basic idea underlying these models is to leverage validated biological information to identify known interactions, hence offering important insights into planning new experiments to validate PPIs from target proteins. The current PPI prediction computational models fall into two main categories: integrated models, which take into account the biological information of proteins retrieved from many sources, and network‐based models, which only rely on PPI network data. Recently, some other attempts have also been made to predict PPI using DL algorithms [33].

**FIGURE 4:**
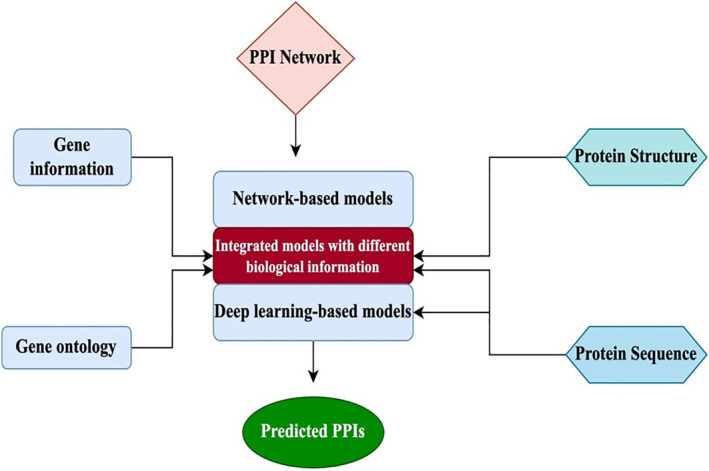
An illustration of the biological knowledge incorporated into several computational models for PPI prediction.

Figure 4 depicts an overall operational process of computational models. Integrated models combine various biological data such as gene information, Gene Ontology, protein structure and protein sequence to predict PPIs. On the other hand, network‐based models and other computational models only use PPI networks for the prediction.

Specific computational models with their advantages and disadvantages are briefly described below.

### 4.1 Network‐Based Computational Models

As the interactome's coverage has grown, network‐based computational models have been created to anticipate missing PPIs by utilising connectivity patterns in a PPI network to characterise existing PPIs. Although social network analysis‐based network‐based link prediction algorithms can be used to tackle the PPI prediction problem, they are unable to capture the connectivity patterns that direct the creation of PPI networks because two proteins interact when one of them is comparable to the other's interacting partners. This means that the connection between two query proteins is highly correlated with the presence of an interaction between them. Protein pairings are to be scored according to their connectivity in the PPI network by network‐based computational models in order to ascertain whether or not these paired proteins interact [34].

### 4.2 Sequence‐Based Computational Models

Amino acids are the basic building blocks of proteins. The primary conformation of a protein is dictated by the arrangement of its amino acids. Many efforts have been made to develop various computational models based on protein sequences, owing to the wealth of relevant data included in them. Before showcasing some example studies in this discipline, we will first explain the mathematical notations related to protein sequence information.

Given an alphabet set *Γ* = {${ϒ}_{i\;}$} consisting of total nΓ different amino acids, a protein sequence S with length $n_{S}$ is represented as *S* = ($s_{t\;}$), where $1\leq S_{t\;}\leq n_{s\;}\;and\;S_{t\;}\epsilon$ *Γ*.

Therefore, a k‐mer segment starting from the position *t* in *s* is denoted as $S_{t,k\;}{=s}_{t\;},s_{t+1\;},\text{…}.,s_{t+k-1\;}$) where $1\leq t\leq n_{s}-k+1.$ Some representative studies in this direction are introduced as follows [34].

### 4.3 Structure‐Based Computational Models

Accurate PPI prediction is made possible by the relevant information that protein structures, in addition to sequences, contain about the biological processes and activities of proteins. PrePPI, a popular structure‐based computational model, shows that three‐dimensional structural information may also be used to predict PPIs more accurately and comprehensively than predictions based on nonstructural information. To achieve this, PrePPI first uses Bayesian statistics to incorporate evidence of both structural and nonstructural interactions. Thus, a Bayesian network can be used to aggregate five empirical ratings and calculate the likelihood of their interaction. The experimental findings demonstrate that PrePPI may detect unanticipated PPIs of substantial biological interest and that its accuracy is on par with high‐throughput trials. PrePPI, however, cannot forecast PPIs for proteins whose three‐dimensional structures have not been identified by experimentation [35].

### 4.4 Genomic‐Based Models

The three primary genomic information sources taken into account by the computational models currently in use for PPI prediction are gene fusion, gene order and phylogenetic relationships. PPIs can be predicted by calculating the functional similarity between proteins using this genetic information. The evolution of gene architecture depends critically on gene fusion. If two proteins are found to have homologues in another genome where they are fused into a single protein, then they interact with each other. To find fusion events in various genomes, Yang [36] created a computational model in this regard. Proteins participating in a fusion event are more likely to interact with one another in this phase. The drawback of gene fusion, however, is that it is impossible to predict protein interactions in cases where fusion events are not identified by a genome sequencing study [37].

Proteins' evolutionary histories are referred to as phylogenetic relationships, and they are frequently displayed within a phylogenetic tree. Similar phylogenetic relationships indicate a functional relationship between proteins, and they are likely to interact to carry out comparable molecular tasks. Phylogenetic profiles are employed to forecast PPIs. Phylogenetic profiles are particularly useful in characterising the co‐evolution history of proteins, and interactions between proteins with similar patterns are highly probable. This work, the first of its kind, shows that PPI prediction can also benefit from comparing the evolutionary profiles of proteins. Unfortunately, due to the limited number of fully sequenced genomes available at the time, very little data were used to create the protein profiles, which reduced their usefulness.

### 4.5 Deep Learning‐Based Computational Models

DL has garnered significant attention from academics in various computational domains, including natural language understanding, ML and image processing, owing to its potent unsupervised feature learning capabilities. Additionally, several attempts have been made to use DL approaches for PPI prediction; these are described in detail below. A deep neural network framework, called DNN‐PPI, is based on characteristics automatically retrieved from protein sequences to examine the overfitting and generalisation of DL models in predicting PPIs. By specifically using the sequences of two interacting proteins as input, DNN‐PPI sequentially feeds the data into the layers of the long short‐term memory neural network, convolution neural network, encoding and embedding neural networks. Subsequently, DNN‐PPI combines the two outputs of the preceding layer into a solitary vector, which is connected as the input of the fully connected neural network. In order to learn the network weights for PPI prediction, DNN‐PPI finally uses the Adam optimiser [36].

Deep learning (DL) has attracted substantial attention across diverse computational domains, including natural language understanding, machine learning and image processing, primarily due to its powerful capability for automatic and unsupervised feature learning. In the context of protein–protein interaction (PPI) prediction, DL methods have emerged as promising alternatives to traditional machine learning approaches, which rely heavily on handcrafted features and domain‐specific heuristics. By learning hierarchical representations directly from protein sequences, DL models can capture complex nonlinear dependencies that are difficult to model using conventional techniques. Several DL‐based frameworks have been proposed for PPI prediction, among which DNN‐PPI represents a notable example. DNN‐PPI is designed to automatically extract discriminative features from raw protein sequences while addressing challenges related to overfitting and generalisation. In this framework, the amino acid sequences of two interacting proteins are used as direct inputs, eliminating the need for manual feature engineering. The model employs a sequential architecture that integrates long short‐term memory (LSTM) networks and convolutional neural networks (CNNs), enabling it to simultaneously capture long‐range sequence dependencies and local motif‐level patterns. Encoding and embedding layers further transform symbolic amino acid representations into dense numerical vectors suitable for neural processing.

After feature extraction, DNN‐PPI concatenates the learnt representations of the two proteins into a single joint feature vector, which is then passed to a fully connected neural network for interaction classification. The use of the Adam optimiser facilitates efficient weight updates and stable convergence during training, enhancing predictive performance. However, although the conceptual architecture of DNN‐PPI is sound, existing descriptions often remain high level, limiting reproducibility and interpretability. Critical implementation details—such as convolutional kernel sizes, stride parameters, activation functions, loss functions and regularisation strategies—are typically omitted or insufficiently explained.

Similar limitations are observed in other DL‐based PPI models, such as PhosIDN, which focuses on identifying phosphorylation‐related interaction patterns. Although these models demonstrate strong predictive performance, their practical utility would be significantly improved by providing clearer methodological explanations. Incorporating flow charts that visually depict data flow across network layers would help readers understand the end‐to‐end architecture. Additionally, including mathematical formulations of the loss function and optimisation objective would clarify how interaction probabilities are learnt. Pseudocode outlining the training and inference pipeline could further enhance transparency and facilitate implementation by other researchers.

**Table:** 

Textual flow chart description (DNN‐PPI)
The workflow of the DNN‐PPI model proceeds as follows:
1. Input layer: Two raw protein amino acid sequences P1P_1P1 and P2P_2P2 are provided as input.
2. Sequence encoding: Each amino acid is converted into a numerical representation using encoding schemes such as one‐hot encoding or embedding vectors.
3. Feature extraction layer A convolutional neural network (CNN) extracts local sequence motifs. A long short‐term memory (LSTM) network captures long‐range dependencies within protein sequences.
4. Feature embedding: Outputs from CNN and LSTM layers are embedded into fixed‐length dense vectors for both proteins.
5. Feature fusion: The embedded representations of P1P_1P1 and P2P_2P2 are concatenated into a single feature vector.
6. Fully connected layer: The fused vector is passed through one or more dense layers to learn interaction‐specific representations.
7. Output layer: A sigmoid‐activated neuron produces the probability of protein–protein interaction.

#### 4.5.1 Input Layer

The DNN‐PPI model begins by accepting two raw protein amino acid sequences, denoted as P1P_1P1 and P2P_2P2. Each sequence represents a protein involved in a potential interaction pair. These sequences are typically composed of standard amino acid symbols and may vary in length. Using raw sequences as input allows the model to learn interaction patterns directly from biological data without relying on manually engineered features.

#### 4.5.2 Sequence Encoding

Because neural networks operate on numerical data, the amino acid sequences are first transformed into numeric representations. Each amino acid is encoded using techniques such as **one‐hot encoding**, where each residue is represented as a binary vector, or **embedding‐based encoding**, where amino acids are mapped to dense, low‐dimensional vectors. This step preserves the biochemical identity of residues while making the sequences suitable for deep learning processing.

#### 4.5.3 Feature Extraction Layer

This stage is responsible for learning informative patterns from the encoded sequences.

- **Convolutional neural network (CNN):** The CNN applies convolutional filters across the sequence to detect local motifs and short‐range residue patterns, such as conserved amino acid segments or functional domains. These local features are crucial for identifying biologically meaningful interaction signals.
- **Long short‐term memory (LSTM) network:** The LSTM captures long‐range dependencies and sequential relationships within protein sequences. It models how distant amino acids influence each other, which is essential for representing global structural and functional characteristics of proteins.

Together, CNN and LSTM complement each other by extracting both local and contextual sequence information.

#### 4.5.4 Feature Embedding

The outputs generated by the CNN and LSTM layers are transformed into **fixed‐length dense vectors**, known as embeddings. These embeddings provide compact and informative representations of each protein, ensuring that variable‐length sequences are mapped into uniform feature spaces suitable for comparison and fusion.

#### 4.5.5 Feature Fusion

In this step, the embedding vectors of P1P_1P1 and P2P_2P2 are concatenated to form a single joint feature vector. This fusion operation enables the model to learn interaction‐specific relationships by jointly considering the characteristics of both proteins rather than analysing them independently.

#### 4.5.6 Fully Connected Layer

The fused feature vector is passed through one or more fully connected (dense) layers. These layers integrate and refine the combined features, learning higher‐level abstractions that are directly associated with protein–protein interaction likelihood. Nonlinear activation functions are typically applied to enhance representational power.

### 4.6 GO‐Based Computational Models

GO‐based computational models quantify the similarities between proteins based on their function, thereby assessing the physiological relevance between pairwise proteins. This approach is driven by the intuition that interacting proteins are more likely to be located in similar locations or participate in similar biological processes. Typically, these computational models aim to create feature vectors for protein pairings, which are subsequently combined with conventional classifiers to predict PPI. A pair of proteins is treated as a text made up of words, where each word is a common GO keyword that these two proteins share. To create feature vectors for protein pairings, each distinct word is considered a feature. Each feature's value is determined by multiplying the relevant term's information content by a coefficient, which stands for the term's weight inside a document. The inherent directed acyclic graph structure of GO is disregarded, despite the experimental findings showing that GO‐based features outperform sequence‐based spectrum count features in terms of performance [36]. Therefore, taking into account all of the links that exist between GO keywords can help to increase prediction accuracy. The similarity results may be overstated or underestimated because the majority of semantic similarity metrics used to evaluate the confidence of PPIs overlook the unbalanced depth in the hierarchy of GO categories as well as the various GO terms associated with cell locations. In light of the uneven depth of biological knowledge representation in various GO hierarchy branches, TCSS has adopted an improved topological clustering semantic similarity. However, in some cases—such as when calculating functional similarity in a broader sense—the use of the similarity function to evaluate PPIs might be overstated.

### 4.7 Computational Models for Large‐Scale PPI Prediction

Currently, less than 20% of the entire interactome's protein interactions are known to exist. The amount and complexity of protein interaction data have grown dramatically along with high‐throughput technology. This presents a new hurdle for large‐scale PPI prediction. Numerous attempts have been made recently in this field. By transforming the hidden internal structures in low‐dimensional latent semantic space, the LDA‐RF model can handle large‐scale datasets and is designed to predict human PPIs explicitly from protein sequences [37]. The scalability of LDA‐RF is limited by the costly inference process of latent Dirichlet allocation, even though random forest performs well on large‐scale prediction tasks. A parallel SVM model relies only on protein sequence data for large‐scale PPI prediction, thereby achieving the goal of efficiently and accurately predicting PPIs [4]. To extract local sequential characteristics from protein sequences, the autocorrelation descriptor approach is first used. Afterwards, distributed SVM classifiers are trained using the MapReduce framework, which allows for a significant reduction in training time. The fact that this model's extraction of local sequential features is not parallelisation‐ready is a productivity hurdle. Table 2 presents a concise overview of various computational models, together with their respective benefits and drawbacks.

**TABLE 2:** Summary of different computational models with their advantages and disadvantages.

Type	Representative model	Description	Advantages	Disadvantages	Reference
Network‐based models	L3	L3 is based on the observation that proteins tend to interact when one protein is similar to the partner of another rather than directly similar to each other.	Captures indirect neighbourhood similarity for PPI prediction.	Unable to predict PPIs between proteins that are distant in the network and do not share neighbours.	[38]
	SpectralLink	SpectralLink employs multiway spectral clustering to capture the topological affinity between proteins.	Considers global structural information of the PPI network.	Many complex structural characteristics of real networks may be ignored.	[39]
	RWS	Random walk similarity (RWS) estimates higher‐order topological similarity between proteins using random walk processes.	Handles sparsity, skewed degree distribution and noise in PPI networks.	The simple cut‐off strategy for retaining edges may reduce robustness.	[40]
Sequence‐based models	VLASPD	Predicts PPIs by analysing variable‐length protein sequence patterns.	Captures sequence motifs of different lengths.	A large number of patterns may affect classifier performance.	[41]
	CD	Predicts interactions assuming protein pairs with similar substitution rates tend to interact.	Provides detailed evolutionary substitution information.	Cannot infer structural interface properties of PPIs.	[42]
Structure‐based models	PrePPI	Predicts PPIs using Bayesian inference combining structural and nonstructural information.	Uses 3D structural information to identify biologically meaningful PPIs.	Cannot predict interactions when protein structures are unavailable.	[43]
Genomic‐based models	Gene fusion method	Detects gene fusion events through sequence comparison to infer protein interactions.	Effective for identifying interactions derived from fused genes.	Cannot detect interactions where fusion events are absent.	[44]
DL‐based models	DNN‐PPI	Uses deep neural networks to automatically learn features from primary protein sequences.	Eliminates the need for manual feature extraction.	Model performance depends on appropriate network architecture design.	[45]
GO‐based models	TCSS	Predicts PPIs based on Gene Ontology semantic similarity while considering uneven biological knowledge across GO branches.	Accounts for imbalance in biological knowledge representation.	Similarity metrics may be exaggerated in certain cases.	[46]
Large‐scale models	LDA‐RF	Combines latent Dirichlet allocation with random forest to extract latent topic features from protein sequences for PPI prediction.	Effective for large‐scale PPI prediction through latent semantic representations.	LDA inference may introduce scalability challenges.	[47]

A comparison of the strengths, weaknesses and applicability of different computational techniques is shown in Table 3.

**TABLE 3:** Comparative analysis of protein–protein interaction prediction methods based on biological question and data availability.

Methods	Strength	Weakness	Applicability
Sequence‐based methods	Simplicity Data availability Direct analysis of protein sequences	Limited context Single‐level analysis May miss structural insights	Identifying interactions based on primary sequence information Comprehensive structural or functional data are lacking. Rapid screening of large datasets
Structure‐based methods	High accuracy Contextual insights Mechanistic understanding of interactions	Data‐intensive Computational cost Requires high‐quality structural data	Understanding the molecular basis of interactions High‐quality structural data are available. Investigating specific binding interfaces and interaction mechanisms
Interaction network‐based methods	Biological context Scalability Global analysis of cellular networks	Data integration Complexity Potential for false positives/negatives due to network properties	Understanding the organisation and dynamics of cellular networks Comprehensive interaction data are available. Systems biology studies and network analysis
Hybrid methods	Combined strengths of sequence, structure and network analysis Improved prediction accuracy	Complexity Data integration Computational cost	Integrative analysis of diverse data sources Comprehensive understanding of interactions combining sequence, structural and network information Research with mixed data types

Table 2 summarises representative computational approaches for protein–protein interaction (PPI) prediction, categorised according to the type of biological information utilised. Network‐based models such as L3, SpectralLink and RWS exploit the topology of PPI networks to infer potential interactions by analysing structural similarities, clustering properties and higher‐order relationships among proteins. These methods are advantageous because they can capture indirect interaction patterns and global network structures; however, they often struggle with sparse networks or proteins that lack sufficient neighbours. Sequence‐based models, including VLASPD and CD, rely on protein sequence information and evolutionary characteristics to predict potential interactions. These approaches are useful when structural data are unavailable, but they may fail to capture complex structural or functional interaction mechanisms. Structure‐based models, represented by PrePPI, incorporate three‐dimensional protein structure information to improve prediction accuracy and identify biologically meaningful interactions, though their applicability is limited when experimentally determined structures are missing. Genomic‐based approaches, such as the gene fusion method, predict interactions by identifying fusion events across genomes, offering insights into functional relationships but being restricted to cases where such events occur. With the advancement of artificial intelligence, deep learning‐based models such as DNN‐PPI automatically learn complex features from protein sequences without manual feature engineering, improving predictive performance but requiring careful architectural design and large datasets. Additionally, GO‐based methods such as TCSS leverage semantic similarity within Gene Ontology annotations to infer functional relationships between proteins, although the uneven distribution of biological knowledge across GO branches can affect accuracy. Finally, large‐scale models such as LDA‐RF combine latent semantic analysis with ensemble learning to handle large biological datasets, though computational complexity may limit scalability. Overall, these approaches demonstrate the evolution of PPI prediction methods from traditional network and sequence analyses to modern data‐driven and deep learning techniques, each offering unique advantages and limitations depending on the availability and quality of biological data.

## 5 Literature Review

The following study reviews various techniques and approaches for predicting protein sequences. Several researchers have studied this field and their findings are explained below.

Hu [33] proposed a DL architecture called DeepTrio. Using previous research [2] as a foundation, DeepTrio was carefully crafted to provide unmatched visual interpretability of the underlying model in addition to enhanced prediction accuracy. Multiple parallel convolution filters are cleverly used in DeepTrio's design and are strategically exploited to extract highly refined characteristics from protein profiles. The model's capacity to identify complex patterns pertinent to PPI is improved by capturing a variety of protein sequence representations through the use of several filters. Weight polarisation, a prevalent problem in neural network training where specific weights dominate the learning process and result in inferior performance, is one of the noteworthy challenges that DeepTrio tackles. To counteract this, DeepTrio includes a masking operation and a single‐protein class, which guarantee balanced weight distributions and encourage more resilient training dynamics. A number of rigorous tests were carried out to objectively validate the effectiveness of DeepTrio, revealing its ability to elucidate the inner workings of paired‐input neural networks and the subtle influence of individual amino acid residues on PPI. The researchers demonstrated DeepTrio's capacity to provide understandable explanations of model predictions through painstaking analysis, thereby providing greater insights into the molecular elements of protein interactions. Notably, DeepTrio's outstanding performance in PPI prediction tasks was highlighted by its astounding highest reported accuracy of 98.12%. This remarkable outcome establishes DeepTrio as a cutting‐edge approach to improving PPI predictions, providing not only enhanced predictive accuracy but also priceless visual interpretations that aid in a more thorough comprehension of the underlying biological principles. In conclusion, the DeepTrio DL architecture offers a synergistic mix of interpretability and predictive capacity, marking a substantial advancement in the field of bioinformatics. DeepTrio is an essential tool for improving our understanding of complicated biological systems because it clarifies the complex links between amino acid residues and protein interactions and provides visual insights into model predictions.

An experiment targeted at improving the performance of convolutional neural network (CNN) models in protein–protein interaction (PPI) prediction tasks was carried out in 2022 by Wang [35]. The work presented sequence‐statistics‐content (SSC), a unique encoding method intended to improve characteristics and lessen the influence of sequence similarity. Utilising a three‐channel formatting strategy, the SSC method combines statistical data obtained from protein sequences with bigram encoding information. The CNN model can access a more varied and complex collection of characteristics by utilising this multichannel representation, which improves its capacity to identify minute patterns in the input data. The encoded sequence data were fed into a two‐dimensional CNN architecture as part of the experimental setup, and spatial characteristics were extracted using two‐dimensional convolutional kernels. To capture more complete representations of protein interactions, this architecture was selected to take advantage of both the sequential and positional information included in protein sequences. The researchers compared their suggested strategy to other approaches already in use in the area and assessed its performance on several datasets. They clarified the effects of various SSC channel combinations on prediction accuracy and model robustness through thorough experimentation and analysis. The results of the study showed how effective the SSC encoding strategy is in enhancing model performance, and they also offered insightful information on the suitability of deep neural networks (DNNs) for PPI prediction tasks. Notably, the method reached the highest reported accuracy of 78.40%, demonstrating its efficacy in raising the bar in this field.

Nadav [35] introduced a model that was pretrained and then evaluated using standards from protein research and sequence encoding. It employs a deep learning architecture inspired by BERT and incorporates numerous advances for representing both global and local protein representations. The ProteinBERT model has around 16 million trainable parameters, compared to 38 million in the TAPE transformer, 110 million in the BERT‐base and 650 million in the ESM‐1b model. The results demonstrated that pretraining with a nonredundant set of proteins whose input and output sequence lengths were encoded by gradually changing sequence lengths enhanced protein modelling ability. ProteinBERT is effective, adaptable and hence generalises to varied sequence lengths, and its competition with state‐of‐the‐art models results in the provision of effective solutions for a variety of protein‐based tasks.

Yang et al. [36] introduced PhosIDN, a revolutionary DL neural network for predicting the site of phosphorylation that extracts and derives combinations of sequences of different proteins and PPI data. In this approach, a subnetwork of features in protein sequences is made for collecting patterns which are local to the interaction and also longer‐range relationships from protein sequences. It utilised a multilayer perceptron with the implementation of a deep neural network capable of exploiting the complicated relationships between protein sequences and PPI characteristics, resulting in a prediction which can be declared as final. The comprehensive experimental results revealed that PhosIDN, which is proposed only, enhances phosphorylation site prediction performance significantly and also performs comparisons with existing general and kinase‐specific phosphorylation site prediction methods. Two subnetworks for feature encoding (SFENet and IFENet) and one subnetwork for heterogeneous feature combining make up the proposed DL architecture of PhosIDN (HFCNet). The findings of the experiment show that PhosIDNSeq consistently performs better as window size rises, achieving AUC values that are on par with or better than DCCNN for each window size. Depending on the protein family or group, the AUC values (%) of PhosIDN using both sequence and PPI information for kinase‐specific phosphorylation site prediction vary from 88% to 97%. The findings show that PhosIDNSeq can transform the original protein sequence into a meaningful representation and that PhosIDN can be used to create a better representation with more discriminant power for differentiating between phosphorylation sites and non‐phosphorylation sites.

Jha [37] used a combination of 43 features for the first time in a PPI prediction task, including evolutionary features, structural features and physiochemical features. The features were compressed using an SAE model, which is normally used for removal of redundant data and noise. Additionally, the comparison of the proposed work was not thorough, as it was compared to only one approach, and not enough work has been done in this area. Highest reported accuracy: 83.55%.

Czibula [48] presented AutoPPI, a binary and supervised classifier, which is used to predict PPI using an ensemble of 2 autoencoders (AEs), one for noninteracting pairs and the other for interacting pairs. The feature vectors used were CT and AC. Three types of neural network (NN) architectures were used for the AEs: joint–joint, siamese–joint and siamese–siamese. The input of the joint–joint architecture is the features of a protein pair and it outputs renovated features. The siamese–joint architecture works the same as the joint–joint architecture and it has a shared structure at the encoder side that compresses the two proteins in a pair into two encodings and a decoder. The siamese–siamese architecture generates a common representation of the two proteins in a pair through elementwise multiplication of their encodings and a shared decoder is used for obtaining the reconstruction of the proteins. In all 3 architectures, the Adam optimiser and ReLU activation function were used. Highest reported accuracy: 97.9%.

Xu [49] proposed GRNN‐PPI, a method for predicting sequence‐based PPIs. This model combined two feature extraction methods, a novel method and AC that covers evolutionary features using a proposed approach which is mutation spectral radius. The obtained fused feature set was then processed with PCA to eliminate noise and redundant data. General regression neural network (GRNN), a memory‐based learning technique with four layers (input, pattern, summation and output), was utilised for classification. When tested against three benchmarks, six different datasets and two PPI networks, GRNN‐PPI performed admirably. The highest recorded accuracy is 99.97%.

Satyajit [50] presented a novel as well as a hybrid approach that combines DNNs and XGB adapted for use in PPI prediction. Through layer‐by‐layer abstraction of the raw data being fed into the XGB classifier, the deep neural network recovers the information that is buried. The accuracy of the fivefold cross‐validation of the datasets for intraspecies interactions between humans, *Saccharomyces cerevisiae* (core subset), *Helicobacter pylori* and *Saccharomyces cerevisiae* is 96.19%, 98.35%, 99.74% and 97.37%, respectively. The accuracy of the datasets for interspecies interactions between human and *Yersinia pestis* and *Bacillus anthracis* is 9. To extract hidden yet valuable information from the properties of unprocessed protein sequences, DNNs are utilised. This technique produced increased accuracy equivalent to CNN‐FSRF outcomes and yielded results comparable to DCT + ROF.

Sledzieski [51] presented the DL method called D‐SCRIPT that was proposed to address the limitations of small training data size and improve generalisation across species. The hypothesis behind D‐SCRIPT was that a well‐designed model structure and favourable input features generate a representation that portrays the behaviour of structural interactions in proteins. To achieve this, a protein embedding was constructed by taking the concept from Belpre and Berger's pretrained model, which included both sequential and structural information about each single protein. The representation obtained was then minimised in dimension in the projection module, which outputs a representation at an abstract level of protein features. For prediction, D‐SCRIPT evaluates the compatibility score of a small subsequence of both protein sequences in the interaction module, followed by a sparse contact map evaluation in the contact module. Lastly, a modified max‐pooling operation was performed on the contact map to identify interaction probability. The results showed improved generalisation and consideration of structural characteristics of interactions.

Wang et al. [34] published a study in which they predicted PPIs by using a novel matrix consisting of sequence descriptors. The feature vector of a protein was created by combining the proposed MOS descriptor, which considers the order relationship of the entire amino acid sequence, with AA classification. A deep neural network (DNN) was used for prediction, and the network parameters were chosen based on the ReLU activation function, cross‐entropy and Adam optimiser, which depicts the cost function. The network depth, width and learning rate were optimised by varying their range. The DNN‐MOS model was trained using AC, CT and LD, and its performance was compared with previous models using benchmark and nonredundant datasets. Highest reported accuracy: 94.3%. The methodology is suitable for situations where it is important to consider the specific order of amino acids in the protein sequences and the time constraints involved in the analysis.

Alalus et al. proposed a new numerical protein mapping process based on an algorithm to predict PPIs and applied it to COVID‐19 using DNNs. The authors set up a dataset due to the scarcity of suitable data and claimed this mapping, which is based on an algorithm, is a first initiative in the field. The algorithm used the AVL tree for fast search processing and balanced properties. The one‐letter code of each amino acid was changed to a numerical form by the use of the AVL tree, which was then compared to existing mapping methods and fed to a DeepBiRNN for classification. The DeepBiRNN had 3 BiRNN layers with a ReLU activation function and 64, 32 and 16 units, followed by flattening, batch normalisation, dropout and 2 FC layers. The resulting performance was favourably convenient for this novel mapping process.

Rachel [52] performed a thorough computational analysis of the SARS‐CoV‐2 interactome with human proteins present in infected HEK 293 cells in order to identify the processes that the virus influences and potential protein binding sites. Using the GoNet algorithm, SARS‐CoV‐interacting human proteins were organised into PPI networks based on 329 Gene Ontology keywords. In addition, unique protein sequence motifs that are anticipated to be significantly affected by SARS‐CoV‐2 were identified. On sets of proteins, functional and motif enrichment analyses were undertaken. From the STRING database, the authors also constructed PPI networks of human proteins that interacted with SARS‐CoV‐2 proteins.

Lu [53] presented the ResPPI algorithm as a novel approach for predicting PPIs based on residual networks. The algorithm uses an embedding method, which is commonly used in natural language processing tasks, to represent the amino acid sequences as vectors. These vectors are then linked together and fed into the residual network (ResNet) for capturing deep features. The residual network, ResNet, is inspired by the successful use of ResNet in other applications. The ResPPI algorithm is composed of 5 residual units, each of which consists of three 2D convolution layers, batch normalisation, a mapping function with a ReLU activation and another convolution layer, in addition, that serves as a timesaver in some cases. The output from the residual units is then fed into a fully connected layer with a softmax function used for binary classification.

Zhang et al. [54] suggested a DL method that enhances the ability of protein interaction site prediction. The authors created a new SLSTM network for implementing the DLPred DL architecture. The model achieved 38.9%, 69.1% and 80.1% in F‐measures, prediction accuracies and AUC values on Dset186; 42.6%, 69% and 81.1% on Dtestset72; and 38.8%, 68.4% and 78.9% in F‐measures, accuracy and AUC on PDBtestset164. The collection of protein sequences with a high percentage of interacting residues for the training dataset, the addition of a new penalisation factor in the loss function and multitask learning PPIS prediction in addition to residue solvent accessibility prediction were three imbalance issues that the model addressed. It has been determined, based on the experimental findings and in comparison to existing state‐of‐the‐art methodologies, that the accuracy of this model is superior.

Yu et al. [55] proposed a new pipeline for PPI predictions which are based on gradient tree boosting (GTB). PsePSSM, PseAAC, AD and RSIV datasets were used. L1‐RLR (L1‐regularised logistic regression) has been used for selecting an optimal feature subset for performing feature extraction. GTB can be employed for predicting binary levels on 4 independent test datasets and 2 network datasets. The GTB classifier is used to close the information gap between the amino acid sequence's informational characteristics and the class label, with the number of iterations set to 1000 and the loss function set to deviation. The results of the experiments indicate that GTB performs better than RF, SVM, KNN and NB. L1‐RLR outperforms PCA, SSDR, mRMR, CMIMs, FA and KPCA in terms of performance. Fivefold cross‐validation was performed on the GTB‐PPI model after it was built, and the results revealed that the model achieved accuracy levels of 90.47% and 95.15% on the datasets for *Helicobacter pylori* and *Saccharomyces cerevisiae*, respectively. The PPI prediction performance has increased because of GTB‐PPI.

Guo and Chen [56] proposed a DL framework to predict PPIs based on the amino acid properties. This framework first creates a feature vector using a descriptor called conjoint AAindex (CAM) modules, which encodes a conjoint amino acid unit of the protein sequence using the amino acid index database. This process is repeated for the entire protein sequence for the generation of a sequence profile. For analysing the conjoint AAindex (CAM) module patterns, multiple dense operators are employed, and a ReLU function is used for introducing nonlinearity. A long short‐term memory (LSTM) layer is added to capture long‐term order dependencies, and the results are calculated using logistic regression. Highest reported accuracy: 92.72%. Use case: This PPI prediction method is suitable in situations where it is important to understand the interactions and protein functions in cellular processes.

Yao [57] predicted PPIs by combining DL with representation learning (RL). The purpose of including RL was to automatically learn patterns from raw data and use the resulting representation in the DL model. The authors proposed a framework named DeepFE‐PPI that leverages the benefits of representation learning to represent data using Res2vec and the benefits of DL by feature extraction with an architecture that is hierarchical and multilayered and PPI classification with a softmax function. The framework has two separate deep neural network modules for feature extraction from a joint module for PPIs classification and two embedding vectors. Highest reported accuracy: 98.71% Use case: The use case of the study is to predict PPIs by combining DL with representation learning. The study aimed at discovering effective features, underlying patterns and mapping of PPIs through a DeepFE‐PPI framework.

Poux [58] proposed the UniProt Knowledgebase (UniProtKB). For this endeavour, it has been mostly utilised. Data from UniProtKB are divided into two groups: reviewed data and unreviewed data. A reviewed dataset including 557,992 proteins is called Swiss‐Prot. The dataset TrEMBL comprises 120,243,849 proteins and is unreviewed. Because the data are reviewed and rectified by user forums and communities, the reviewed dataset has often been utilised for protein function prediction. Numerous species can be found in the Swiss‐Prot dataset. In this study, only human organisms were taken into account, and protein data from the human enzyme class were retrieved for protein categorisation. The provided Table 4 is a summary of the reviewed literature from different authors. Their methods and the results of their work are also described below.

**TABLE 4:** Summary of literature review.

Authors	Techniques	Outcomes
Alakus and Turkoglu [59]	DNNs	Applied a novel algorithm‐based protein numerical mapping process to predict PPIs on COVID‐19; results showed favourable performance.
Yu et al. [55]	Gradient tree boosting (GTB) + L1‐regularised logistic regression (L1‐RLR)	On *Saccharomyces cerevisiae* and *Helicobacter pylori*, GTB‐PPI attained accuracies of 95.15% and 90.47% using fivefold cross‐validation; improved PPI prediction performance.
Zhang et al. [54]	DL, SLSTM network	Experimental results show the model outperforms current state‐of‐the‐art methods in accuracy.
Czibula et al. [48]	Neural network	Siamese–siamese architecture generates shared protein representations via elementwise multiplication and a common decoder; highest reported accuracy: 97.9%.
Guo and Chen [56]	DL	The deep learning framework achieved 92.72% accuracy; suitable for understanding protein connections and roles in biological processes.
Yang et al. [36]	Deep neural network (DNN)	PhosIDN with sequence + PPI information achieved AUC values between 88% and 97%, improving kinase‐specific phosphorylation site prediction.
Hu et al. [33]	DL	DeepTrio demonstrated 98.12% accuracy and effectively explained pairwise‐input NNs and amino acid residue effects on PPIs.
Jha et al. [37]	SAE model	Predicted PPI using 43 compressed features with SAE; highest reported accuracy: 83.55%.
Lu et al. [53]	Neural networks	The ResPPI algorithm predicted PPIs using residual networks; highest reported accuracy: 96.69%.
Nadav et al. [35]	DL	Pretraining on nonredundant protein sets with varied sequence lengths gradually improved ProteinBERT modelling and sequence prediction performance.
Rachel et al. [52]	GoNet algorithm	Computational analysis of SARS‐CoV‐2 interactome in HEK 293 cells identified viral‐influenced processes and probable protein binding sites.
Satyajit et al. [50]	Deep neural network + XGB	The DNN‐XGB model achieved 98.50% and 97.25% accuracy for intraspecies PPI prediction and reduced time and computational load.
Sledzieski et al. [51]	DL	The DL D‐SCRIPT method improved generalisation and captured structural interaction characteristics.
Wang et al. [34]	DL	DNN‐MOS trained on AC, CT and LD; considered amino acid order and time constraints; highest reported accuracy: 94.43%.
Wang et al. [25]	Concurrent neural networks (CNN)	SSC channel combination analysis provided insights for DNNs in PPI prediction; highest reported accuracy: 78.40%.
Xu et al. [49]	General regression neural network	The GRNN‐PPI model predicted sequence‐based PPIs with highest reported accuracy of 99.97%.
Yao et al. [57]	DL with feature embedding	The DeepFE‐PPI framework discovered effective features and mappings; highest reported accuracy: 98.71%.

### 5.1 Classification of Existing Approaches for Protein–Protein Interaction Prediction

Research on protein–protein interaction (PPI) prediction has evolved through several methodological categories, including sequence‐based learning, feature engineering approaches, network‐based models and deep learning‐driven architectures. Early studies primarily relied on handcrafted features derived from amino acid properties, evolutionary profiles and physicochemical descriptors. For example, Jha [60] utilised a combination of 43 heterogeneous features including structural, evolutionary and physicochemical characteristics to predict PPIs, whereas dimensionality reduction using stacked autoencoders was applied to eliminate redundancy and noise. Similarly, Xu [49] proposed GRNN‐PPI, which integrates mutation spectral radius and autocovariance features before classification using a general regression neural network. Feature‐based machine learning pipelines such as GTB‐PPI also emerged, combining descriptors such as PsePSSM, PseAAC, AD and RSIV with gradient tree boosting classifiers. As research progressed, deep learning approaches became more prominent because of their ability to automatically extract hierarchical representations from protein sequences. Several deep neural network architectures have been proposed for this purpose. For instance, Guo and Chen [56] developed a deep learning model using conjoint AAindex modules combined with LSTM layers to capture sequential dependencies in protein sequences. Yao [57] introduced the DeepFE‐PPI framework, which integrates representation learning with deep neural networks to automatically learn meaningful embeddings for protein sequences. Later studies expanded these deep learning approaches by incorporating convolutional neural networks and residual architectures. Lu [53] introduced ResPPI, which applies residual networks to capture complex sequence representations. Additionally, Wang [34] developed the DNN‐MOS framework, which integrates amino acid order descriptors with deep neural networks for improved sequence representation. Another important category includes hybrid architectures that combine multiple learning paradigms. For example, Satyajit [50] combined deep neural networks with XGBoost to extract hidden features from raw protein sequences before classification. Similarly, Czibula [48] proposed AutoPPI, which utilises ensembles of autoencoders to distinguish interacting and noninteracting protein pairs. In recent years, transformer‐based architectures inspired by natural language processing have also been explored. ProteinBERT [35] introduced a large‐scale pretraining strategy that captures both local and global contextual relationships within protein sequences. Parallel to sequence‐based models, specialised deep learning frameworks such as PhosIDN [36] were developed to integrate sequence features and interaction network information simultaneously. Recent advancements have also focused on improving interpretability and biological insight. For example, Hu [33] proposed the DeepTrio architecture, which integrates multiple parallel convolution filters to enhance feature extraction while providing visual interpretability of model predictions. Collectively, these studies demonstrate that PPI prediction research spans multiple methodological paradigms, including classical machine learning, feature‐driven models, hybrid learning frameworks and modern deep learning architectures. Each category contributes different perspectives to the problem of modelling protein interactions, reflecting the increasing complexity and diversity of computational approaches in bioinformatics.

### 5.2 Comparative Overview of Computational Methods in PPI Prediction

A comparative perspective of existing studies reveals differences in model architectures, feature representation strategies and evaluation methodologies used for PPI prediction. Traditional machine learning approaches largely depend on handcrafted features derived from biological sequences and domain knowledge. Methods such as GRNN‐PPI [49] and GTB‐PPI employ statistical descriptors and evolutionary information extracted from protein sequences before applying machine learning classifiers. These models often rely on dimensionality reduction techniques such as PCA or feature selection methods such as L1‐regularised logistic regression to improve performance and remove redundant information. In contrast, deep learning approaches aim to eliminate the need for manual feature engineering by automatically learning representations from raw sequence data. For instance, DeepFE‐PPI [57] combines representation learning with deep neural networks to discover hidden patterns in protein sequences. Similarly, Guo and Chen [56] proposed a framework using LSTM networks to capture long‐range dependencies in amino acid sequences. CNN‐based architectures also play a significant role in PPI prediction tasks. ResPPI [53] utilises residual convolutional layers to learn hierarchical representations of protein sequences, whereas the SSC‐CNN framework proposed by Wang [25] introduces a three‐channel encoding scheme to combine statistical sequence information with bigram representations. These architectures allow models to capture both spatial and sequential characteristics of protein sequences. Hybrid methods represent another category of computational techniques that combine deep learning with ensemble learning algorithms. The hybrid DNN‐XGBoost approach proposed by Satyajit [50] leverages the feature extraction capability of deep neural networks together with the classification power of gradient boosting methods. Similarly, AutoPPI [48] uses ensemble autoencoders to learn representations for interacting and noninteracting protein pairs. Transformer‐based models further extend this comparative landscape by incorporating attention mechanisms to model complex relationships within protein sequences. ProteinBERT [35] adopts a pretraining strategy similar to language models and demonstrates improved generalisation across multiple protein‐related tasks. Additionally, models such as PhosIDN [36] integrate heterogeneous biological data sources, including sequence features and protein interaction networks, to improve predictive accuracy. Although performance metrics reported across studies vary depending on dataset and experimental settings, many models achieve high accuracy levels exceeding 90%, with some approaches reporting results above 98%. However, direct comparisons remain challenging due to differences in datasets, evaluation protocols and feature extraction techniques. Despite these variations, the comparative overview highlights that modern PPI prediction models increasingly emphasise automated feature learning, integration of multiple biological data sources and scalable deep learning architectures.

### 5.3 Common Limitations in Existing PPI Prediction Studies

Although significant progress has been achieved in computational PPI prediction, several common limitations remain evident across existing studies. One of the primary challenges involves the availability and quality of training datasets. Many models rely on curated protein interaction databases such as UniProtKB [58], which contain both reviewed and unreviewed protein sequences. Although reviewed datasets such as Swiss‐Prot provide high‐quality annotations, the overall availability of experimentally validated interactions remains limited compared to the enormous number of possible protein pairs. This limitation can affect model generalisation and may lead to biases in training data. Another widely reported limitation relates to feature representation. Traditional machine learning approaches often depend on handcrafted descriptors such as evolutionary profiles, amino acid composition or physicochemical features. Although these descriptors capture useful biological information, they may fail to represent complex structural and contextual relationships within protein sequences. Even in deep learning models, the representation of long‐range dependencies and structural interactions can remain challenging. For example, models such as GRNN‐PPI or GTB‐PPI rely heavily on predefined feature extraction processes, which may introduce redundancy or noise in the input data. Similarly, autoencoder‐based frameworks such as AutoPPI require careful architectural design to avoid information loss during feature compression. Another limitation concerns model interpretability. Although deep learning architectures such as CNNs, LSTMs and transformer‐based models achieve high prediction accuracy, they often function as black‐box systems with limited biological interpretability. Although approaches such as DeepTrio attempt to address this issue by providing visual interpretation of model predictions, the challenge of explaining deep learning decisions remains an open research problem. Additionally, many models focus solely on protein sequence information and do not incorporate complementary biological data sources such as structural information, functional annotations or interaction networks. Methods such as PhosIDN attempt to integrate multiple data types; however, heterogeneous data integration remains complex due to differences in data formats and reliability. Another issue relates to evaluation and benchmarking. Many studies report high accuracy values but compare their models with only a limited number of baseline methods or datasets, making it difficult to assess general performance across different biological contexts. Furthermore, models trained on a specific organism or dataset may not generalise well to other species or experimental conditions. Finally, computational complexity and scalability represent additional concerns, particularly for deep learning architectures with large numbers of parameters or complex training procedures. These limitations indicate that although current models provide valuable tools for PPI prediction, several methodological and practical challenges remain to be addressed.

### 5.4 Research Gap and Motivation for the Proposed Approach

The review of existing literature highlights several research gaps that motivate further investigation into improved computational frameworks for PPI prediction. One significant gap relates to the integration of multiple sources of biological information. Many existing studies focus primarily on sequence‐based representations, whereas structural, evolutionary and network‐level information are often considered separately rather than within a unified framework. This separation can limit the ability of models to capture the full complexity of protein interactions, which are influenced by multiple biological factors simultaneously. Another gap concerns the balance between predictive accuracy and interpretability. Deep learning architectures such as CNNs, LSTMs and transformer models have demonstrated strong predictive performance; however, their internal decision processes remain difficult to interpret biologically. Although some models attempt to address interpretability through visualisation techniques or attention mechanisms, the development of computational methods that simultaneously provide high accuracy and meaningful biological insights remains an open research challenge. Additionally, many existing approaches rely heavily on large training datasets, which may not always be available for certain organisms or protein families. Models trained on limited datasets may suffer from overfitting or reduced generalisation ability when applied to unseen proteins or cross‐species prediction tasks. Another limitation involves the limited evaluation diversity observed in several studies, where models are tested on a small number of benchmark datasets without comprehensive cross‐dataset validation. This restricts the ability to assess model robustness under varying biological conditions. Furthermore, feature redundancy and noise remain critical issues in many machine learning pipelines. Although dimensionality reduction techniques such as PCA and autoencoders are commonly applied, the optimal combination of feature extraction and representation learning methods continues to be an active area of research. Recent studies also highlight the potential benefits of combining representation learning with advanced neural architectures, suggesting opportunities for hybrid frameworks that integrate deep learning, feature optimisation and biological domain knowledge. These observations indicate that there is still considerable scope for developing improved computational approaches capable of handling heterogeneous biological data, improving generalisation across datasets and providing more interpretable insights into protein interaction mechanisms. Addressing these gaps can contribute to more reliable and biologically meaningful prediction systems, thereby supporting advancements in computational biology and bioinformatics research.

## 6 Literature Comparison

A comparative analysis for comparing the methods based on their experimental results has been done, and the accuracies have been shown in Table 5 below.

**TABLE 5:** Qualitative comparison with prior studies rather than a direct experimental comparison.

Authors	Techniques	Outcomes
Hu et al. [33]	DL	Highest reported accuracy: 98.12%.
Jha et al. [60]	SAE model	Highest reported accuracy: 83.55%.
Czibula et al. [48]	Neural network	Highest reported accuracy: 97.90%.
Xu et al. [49]	General regression neural network	Highest reported accuracy: 99.97%.
Satyajit et al. [50]	Deep neural network (DNN) + XGB	Highest reported accuracies: 98.50% (DNN), 97.25% (XGB).
Yu et al. [55]	Gradient tree boosting (GTB) + L1‐regularised logistic regression (L1‐RLR)	Highest reported accuracies: 95.15% (GTB), 90.47% (L1‐RLR).
Yao et al. [57]	DL with feature embedding	Highest reported accuracy: 98.71%.

**FIGURE 5:**
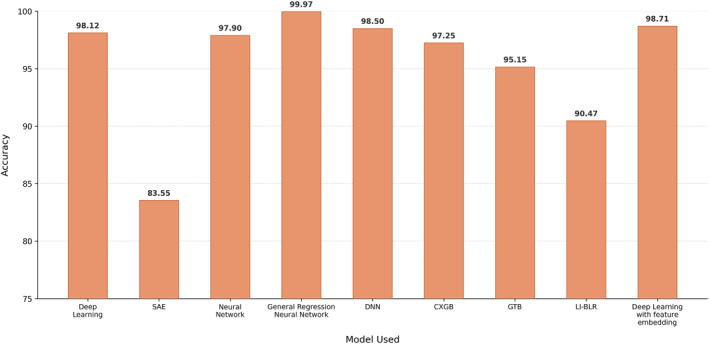
Comparative accuracy of different computational models for protein–protein interaction prediction.

Figure 5 presents a comparative performance analysis of multiple machine learning and deep learning models based on classification accuracy, highlighting clear differences in predictive capability across approaches. The results indicate that advanced neural architectures consistently outperform conventional and shallow models. Among all evaluated techniques, the general regression neural network achieves the highest accuracy of **99.97%**, demonstrating its strong capability to model complex nonlinear relationships in the data. This is closely followed by deep learning with feature embedding (**98.71%**), DNN (**98.50%**) and standard deep learning (**98.12%**), underscoring the effectiveness of deep architectures, particularly when enriched with informative feature representations. The neural network model also performs competitively with an accuracy of **97.90%**, whereas the cXGB model achieves **97.25%**, indicating robust learning performance among ensemble and gradient‐based methods. In contrast, traditional and comparatively simpler models exhibit lower predictive accuracy. The GTB model records **95.15%**, reflecting moderate effectiveness but reduced adaptability to intricate data patterns. L1‐RLR further declines to **90.47%**, suggesting limitations in handling high‐dimensional or nonlinear feature spaces. The SAE model shows the lowest accuracy at **83.55%**, indicating insufficient representational capacity for the given task. Overall, the figure clearly illustrates that deep learning‐based models, particularly those integrating feature embedding and advanced neural regression mechanisms, provide superior accuracy compared to conventional machine learning approaches. This performance gap highlights the importance of deep feature extraction, nonlinear learning capability and model complexity in achieving high predictive accuracy. Consequently, Figure 5 substantiates the advantage of modern deep learning frameworks for accurate and reliable classification, reinforcing their suitability for complex, data‐intensive applications over traditional methods.

This study revealed the reported accuracy values of different techniques as follows: DL achieved the highest reported accuracy of 98.12%. The average reported accuracy of SAE models was 83.55%. The reported accuracy values for neural networks averaged 97.90%. General regression neural networks had reported accuracy values averaging 99.97%. The average reported accuracy for DNNs and extreme gradient boosting was 98.50% and 97.25%, respectively. Gradient tree boosting had a reported accuracy value of 98.17%. Among all these techniques, the general regression neural network has outperformed other methods with the highest accuracy percentage: Then, DL with feature embedding has the second highest accuracy and DNNs have the third highest accuracy, whereas the SAE model has the lowest accuracy and the L1‐regularised logistic regression has the second lowest accuracy.

## 7 Expanded Comparative Discussion: Limitations of Accuracy‐Centric Evaluation in PPI Prediction

In this section, the authors have provided a clear idea about the proposed methodology.

Pseudocode 1: The proposed model for predicting protein interactions using DL.

**Step 1**: DefineBasicAminoAcidBank()

Define a set containing the 20 basic amino acids.

**Step 2**: CollectAndStoreProteinInteractionData()

Gather experimental data on PPIs from wet laboratory tests.

Store the collected data in a database for further use.

**Step 3**: TrainDeepLearningModel()

Train a deep learning model using autoencoders and decoders.

The model is trained with protein interaction data as inputs, which are retrieved from the database.

**Step 4:** DivideDataset(training_percentage, testing_percentage)

Divide the dataset into two subsets: a training set and a testing set.

The training set contains 70%–80% of the full dataset, and the testing set contains 30%–20%.

**Step 5:** ValidateModel()

Validate the trained model's performance using a separate validation dataset.

This dataset is distinct from the training and testing datasets to ensure unbiased evaluation.

**Step 6:** PackageModelForDeployment()

Package the trained model along with its functional dependencies.

Prepare the model for deployment in real‐world applications.

Here,

- DefineBasicAminoAcidBank(): A is defined as a set containing the 20 basic amino acids.
- CollectAndStoreProteinInteractionData(): D represents a matrix containing the PPI data.
- TrainDeepLearningModel(): The model is trained using input data X and output data Y.
- DivideDataset(training_percentage, testing_percentage): The datasets X and Y are divided into training and testing sets based on the specified percentages.
- ValidateModel(): The model's performance is evaluated, resulting in performance metrics.
- PackageModelForDeployment(): The trained model is packaged for deployment.

This section elaborates on the step‐by‐step methodology for PPIs using DL techniques. The approach focuses on designing a comprehensive pipeline starting from defining basic biological principles to deploying a functional model.

**Step 1:** The basic amino acid bank is defined as a collection of the 20 standard amino acids that constitute the fundamental components of proteins. This stage will create a core framework by offering a common reference for encoding protein sequences in later data preparation and modelling phases. This methodology will enhance interoperability between computational methods and biological data, ensuring that the dataset consistently reflects amino acids.

**Step 2:** Experimental data regarding PPIs will be obtained via wet laboratory tests. The unprocessed interaction data will be kept in a database optimised for efficient retrieval and processing. The data generally encompass interaction scores, binding affinities and additional biological parameters. Extensive experimental techniques, including yeast two‐hybrid systems and mass spectrometry, will be employed to acquire these data. Data pretreatment methods, such as normalisation, outlier identification and missing value management, will be implemented to guarantee quality. The structured data will be arranged in a matrix *D*, with rows denoting proteins and columns representing interaction features. This matrix will function as the input for the training and evaluation of the deep learning model.

**Step 3:** A deep learning model will be constructed and trained to utilise autoencoders and decoders to reveal the intricate patterns inherent in PPI data. The model will process input data *X* and produce output data *Y*, which are derived from the database. The autoencoder will convert the high‐dimensional interaction data into a lower‐dimensional latent space, extracting essential features that effectively reflect protein interactions. The decoder will rebuild the original interaction data from these latent properties and will forecast new interactions or validate current interactions. During training, loss functions such as mean squared error (MSE) or binary cross‐entropy will be minimised to enhance model performance, whereas optimisation techniques such as Adam or RMSProp will be employed to adjust the model's weights. To mitigate overfitting and enhance generalisation, regularisation techniques, such as dropout, will be integrated into the model's training regimen.

**Step 4:** The dataset will be partitioned into training and testing groups according to designated percentages. Generally, 70%–80% of the data is designated for training, whereas the remaining 20%–30% is allotted for testing. The training set (X_train_), (Y_train_) will be utilised to optimise model weights, whereas the testing set (X_test_),(Y_test_) will assess the model's generalisability to novel data. Data partitioning will be conducted either randomly or through stratified sampling to provide equitable representation.

**Step 5:** The validation of the trained model will utilise a separate validation dataset that is excluded from both the training and testing sets. This phase will evaluate the model's resilience and guarantee an impartial performance assessment. The evaluated metrics will encompass accuracy, measuring the percentage of correctly predicted interactions; precision and recall, assessing the model's capacity to accurately identify true interactions; the F1‐score, balancing precision and recall for a thorough performance evaluation; and the ROC‐AUC curve, analysing the trade‐off between true positives and false positives. Furthermore, k‐fold cross‐validation will be utilised to enhance the model's consistency assessment across various data partitions.

**Step 6:** The trained model and its dependencies will be packaged for deployment. This approach will involve the creation of a functioning module that can be seamlessly integrated into practical applications. The packaging process will entail exporting the trained model as a serialised file (e.g., .h5 or .pth), including preparation scripts for encoding protein sequences and interaction data, and developing APIs for a smooth interface with hospital systems, laboratories or research tools. Deployment considerations will include assessing interoperability with cloud or on‐premise computing systems and incorporating real‐time data processing capabilities to support large‐scale applications.

**FIGURE 6:**
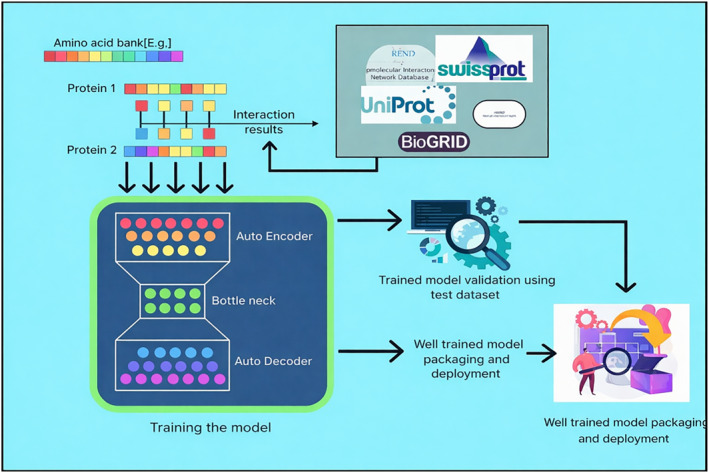
The proposed model for predicting protein interactions using deep learning.

Figure 6 illustrates the suggested DL model for predicting protein interactions.

### 7.1 Comparative Analysis and Methodological Considerations in Protein–Protein Interaction Prediction

Protein–protein interaction (PPI) prediction has evolved from traditional experimental and statistical approaches to sophisticated machine learning‐ and deep learning‐based models. Early computational studies relied on handcrafted sequence descriptors, evolutionary profiles and classical classifiers such as support vector machines, random forests and extreme learning machines, demonstrating moderate predictive capability under controlled settings [2, 9, 61, 62, 63]. Although these approaches provided important insights into sequence‐level interaction patterns, their performance was strongly influenced by feature engineering choices and dataset characteristics.

With the advancement of deep learning, convolutional neural networks (CNNs), recurrent neural networks (RNNs), autoencoders and hybrid architectures have significantly improved PPI prediction accuracy by learning hierarchical and latent representations directly from protein sequences [28, 48, 50, 60]. More recent models, including transformer‐based protein language models such as ProteinBERT and large‐scale unsupervised representations trained on millions of sequences, have further enhanced predictive performance and generalisation capabilities [30, 35]. In parallel, structure‐aware approaches and resources such as AlphaFold and the AlphaFold Protein Structure Database have reshaped the landscape of protein interaction and function prediction by providing highly accurate structural information [14, 15].

However, although many studies report high accuracy values—often exceeding 90%—direct comparison across these studies remains challenging. Existing methods differ substantially in terms of benchmark datasets (e.g., UniProt, STRING, BioGRID, virus–host interaction datasets), sequence redundancy filtering strategies, class balance and negative sample generation [3, 17]. Furthermore, evaluation metrics vary widely, with some studies emphasising accuracy, whereas others prioritise precision, recall, F1‐score, MCC or AUC, each reflecting different aspects of model performance [1, 5]. Differences in experimental conditions—including cross‐validation schemes, train–test splits, preprocessing pipelines and computational resources—further limit the fairness of direct numerical comparisons.

Consequently, reported accuracies should be interpreted as **indicative rather than strictly comparable** across studies. Although deep learning‐based approaches consistently demonstrate superior representation learning and predictive potential, a unified benchmarking framework with standardised datasets, evaluation protocols and reporting metrics is still lacking. Recent survey and review studies emphasise the necessity of reproducible experimental settings and fair evaluation practices to enable meaningful assessment of methodological progress in PPI prediction [17, 26].

In this context, this work positions its contributions not merely in terms of absolute accuracy improvement but also through architectural design choices, representation learning strategies and robustness across evaluation metrics under consistent experimental settings. Future research directions include cross‐dataset validation and standardised benchmarking to ensure more reliable and generalisable comparisons among PPI prediction models.

The prediction of protein–protein interactions (PPIs) has witnessed substantial methodological advancement, transitioning from rule‐based and statistical learning approaches to deep learning and large‐scale representation learning models. Classical approaches primarily relied on sequence‐derived descriptors, evolutionary information and physicochemical properties combined with shallow classifiers, offering interpretability but limited scalability and generalisation [3, 4, 61]. These early models often demonstrated reasonable accuracy under narrowly defined experimental settings; however, their performance was highly sensitive to feature construction, dataset size and noise inherent in biological interaction data.

The emergence of deep learning frameworks—including CNNs, RNNs, stacked and denoising autoencoders, and hybrid architectures—has significantly improved the ability to capture nonlinear and long‐range dependencies within protein sequences [28, 48, 60]. CNN‐based models effectively extract local interaction motifs, whereas recurrent and attention‐based mechanisms model sequential dependencies critical to interaction prediction [10, 33, 54]. Furthermore, ensemble and hybrid strategies integrating gradient boosting or multiple neural components have demonstrated performance gains and improved robustness [50].

More recently, transformer‐based protein language models trained on massive sequence corpora have reshaped the PPI prediction paradigm. Models such as ProteinBERT and large‐scale unsupervised representations leverage contextual embeddings that implicitly encode structural, functional and evolutionary information, often rivalling structure‐based approaches [30, 35]. Complementary breakthroughs in protein structure prediction, most notably AlphaFold and its large‐scale database, have further enabled structure‐aware interaction modelling, bridging the gap between sequence‐based and structure‐based PPI prediction [14, 15]. Despite these advances, comparative analyses across studies frequently rely on reported accuracy values as the primary performance indicator. This practice introduces several methodological limitations. First, datasets used for PPI prediction vary significantly in origin, scale and biological context, including human–human, host–pathogen and virus–host interaction networks [6, 52]. Differences in redundancy reduction thresholds, sequence similarity filtering and negative sample construction strategies can drastically affect model outcomes, often inflating accuracy in artificially balanced or simplified datasets. Second, evaluation protocols differ widely across studies. Although some studies adopt k‐fold cross‐validation, others rely on random hold‐out splits or independent test sets, each offering different insights into model generalisation [5]. The lack of standardised metrics further complicates comparison, as accuracy alone fails to capture class imbalance, false‐positive costs or decision boundary reliability. Metrics such as Matthews correlation coefficient (MCC), area under the ROC curve (AUC) and F1‐score provide more comprehensive performance assessment but are inconsistently reported [17].

Third, experimental conditions—including preprocessing pipelines, sequence encoding schemes, regularisation strategies and optimisation techniques—vary considerably. Architectural enhancements such as batch normalisation, dropout variants and adaptive learning rate schedulers significantly influence convergence behaviour and final performance [24, 29, 64]. Hardware and software configurations, including GPU utilisation and parallel computing frameworks, also impact training efficiency and reproducibility, yet are often under‐reported. As emphasised in recent comprehensive surveys, the absence of unified benchmarking frameworks limits the interpretability of performance gains reported in the literature [1, 17]. Consequently, reported accuracy improvements should be interpreted with caution, as they may reflect dataset‐specific optimisations rather than true methodological superiority. A more rigorous evaluation paradigm—incorporating standardised datasets, multiple evaluation metrics, cross‐dataset validation and transparent reporting—is essential for fair and reproducible comparison. Within this context, the contributions of this study should be understood not merely as incremental accuracy improvements but also as advancements in representation learning, architectural robustness and consistent evaluation under controlled experimental conditions. By addressing known sources of bias and variability in PPI prediction, this work aims to provide more reliable insights into model effectiveness and generalisability, aligning with emerging best practices in computational biology and bioinformatics.

### 7.2 Insufficiency of Technical Detail and Reproducibility Concerns in Existing PPI Models

Although recent studies on protein–protein interaction (PPI) prediction report notable performance improvements, many lack the level of technical detail required for reproducibility and meaningful methodological comparison. Several studies provide high‐level architectural descriptions—such as the use of convolutional, recurrent, autoencoder‐based or transformer‐based models—without specifying critical implementation details, including layer configurations, kernel sizes, activation functions, embedding dimensions and parameter counts [10, 33, 60]. As a result, replicating these models or fairly benchmarking them against alternative approaches becomes challenging. Moreover, essential information regarding data preprocessing and representation learning is often insufficiently documented. Sequence encoding strategies—such as physicochemical property mapping, evolutionary profiles, spectral descriptors or contextual embeddings—play a decisive role in PPI prediction performance yet are frequently described only conceptually, without precise mathematical formulation or parameter settings [9, 35, 63]. In several cases, details on redundancy removal thresholds, negative sample generation and class balancing techniques are omitted, despite their substantial influence on evaluation outcomes. Training protocols also suffer from under‐specification across the literature. Key hyperparameters, including learning rates, batch sizes, optimiser choices, regularisation strategies and early stopping criteria, are inconsistently reported or entirely absent [24, 29, 64]. Similarly, variations in loss functions, convergence criteria and initialisation schemes are rarely discussed, limiting insight into model stability and convergence behaviour. Without this information, reported performance gains may reflect implementation‐specific tuning rather than inherent architectural advantages.

Additionally, reproducibility is further constrained by the lack of transparency regarding experimental environments. Hardware configurations, software frameworks, library versions and random seed settings are often omitted, despite their known impact on deep learning performance and training dynamics [17, 53]. The absence of publicly available code and standardised evaluation pipelines exacerbates these challenges, preventing independent validation and robust comparison. Consequently, although the literature demonstrates rapid methodological progress in PPI prediction, the superficial technical reporting limits the ability to assess true advances and generalisability. Addressing these gaps requires comprehensive disclosure of model architectures, data preprocessing steps, training protocols and experimental settings. In line with emerging best practices in computational biology and machine learning, detailed and transparent reporting is essential to ensure reproducibility, facilitate fair comparison and accelerate reliable progress in protein–protein interaction prediction research.

Table 6 provides a comprehensive overview of the proposed MCONet architecture, detailing its core components, parameter settings and training configurations to address reproducibility concerns commonly observed in prior PPI prediction studies. The model begins with an input representation module that encodes protein sequences into fixed‐length numerical vectors using standardised sequence encoding schemes, ensuring consistency across samples and datasets. This encoding facilitates effective learning of biochemical and sequential patterns essential for interaction prediction.

**TABLE 6:** Architectural and training specifications of the proposed MCONet model.

Module	Component	Specification/description
Input layer	Sequence input	Fixed‐length encoded protein sequences
Encoding scheme	Sequence representation	Numerical encoding of amino acid sequences (standardised across datasets)
Convolution block 1	Conv1D layer	64 filters, kernel size = 3, stride = 1
	Activation	ReLU
	Batch normalisation	Applied after convolution
	Max pooling	Pool size = 2
Convolution block 2	Conv1D layer	128 filters, kernel size = 5
	Activation	ReLU
	Batch normalisation	Applied
	Max pooling	Pool size = 2
Multichannel feature learning	Parallel convolution paths	Multiple receptive fields for diverse feature extraction
Regularisation	Dropout	Dropout rate = 0.5
Flatten layer	Feature flattening	Converts feature maps to a 1D vector
Fully connected layer 1	Dense layer	256 neurons, ReLU activation
Fully connected layer 2	Dense layer	128 neurons, ReLU activation
Output layer	Dense layer	1 neuron, sigmoid activation
Loss function	Binary cross‐entropy	For interaction versus noninteraction classification
Optimiser	Adam	Learning rate = 0.001
Batch size	Training batch	32
Epochs	Maximum epochs	100 (with early stopping)
Evaluation metrics	Performance measures	Accuracy, precision, recall, F1‐score, MCC
Implementation environment	Platform	Python (TensorFlow/Keras)

The convolutional feature extraction block employs multiple one‐dimensional convolutional layers with carefully selected kernel sizes to capture local interaction motifs and residue‐level dependencies. Each convolutional layer is followed by batch normalisation and nonlinear activation functions, which stabilise training and mitigate internal covariate shift. Max‐pooling layers are incorporated to reduce dimensionality while preserving salient features, thereby improving computational efficiency and generalisation. To model long‐range dependencies and contextual relationships between amino acid residues, MCONet integrates a multichannel processing mechanism, allowing parallel feature learning across diverse receptive fields. Regularisation strategies, including dropout layers, are applied to prevent overfitting and enhance robustness, particularly when handling imbalanced or noisy biological datasets. The extracted features are subsequently flattened and passed to fully connected layers that perform high‐level abstraction and decision‐making. The training configuration outlined in Table 6 specifies the optimiser, learning rate, batch size, loss function and number of training epochs. These parameters are selected based on empirical validation to ensure stable convergence and optimal performance. The inclusion of early stopping criteria further prevents overtraining, whereas consistent random seed initialisation enhances experimental reproducibility. By explicitly documenting architectural choices, hyperparameters and training conditions, Table 6 ensures transparency and enables fair comparison with existing PPI prediction models. This level of technical detail directly addresses limitations in prior studies, where insufficient reporting hindered reproducibility and meaningful benchmarking, thereby strengthening the methodological rigour of the proposed MCONet framework. It conceptually illustrates the major shortcomings observed in several protein–protein interaction (PPI) prediction studies, particularly the failure to clearly identify genuine research gaps and propose actionable directions for methodological advancement. The figure highlights four critical dimensions: overlooked research gaps, lack of innovation pathways, limited practical guidance and absence of future research roadmaps. Despite reporting high predictive accuracies, many studies do not adequately analyse unresolved challenges such as dataset bias, negative sample construction, scalability, cross‐species generalisation and reproducibility constraints. The visualisation emphasises that without explicit identification of these gaps, proposed models often remain incremental rather than transformative. The absence of innovation pathways indicates that architectural modifications are frequently presented without justification grounded in biological relevance or comparative experimental analysis. Additionally, limited practical guidance restricts the ability of researchers to translate methodological contributions into reproducible implementations or real‐world biological applications. The lack of clearly defined future directions further hampers cumulative progress in PPI prediction research. Figure 6 underscores the need for studies to move beyond performance‐centric reporting and instead adopt a gap‐driven research perspective. Explicitly articulating open challenges, methodological limitations and concrete future strategies is essential for advancing robust, interpretable and generalisable PPI prediction frameworks. This figure motivates the adoption of standardised benchmarks, transparent reporting and biologically informed model design as key directions for future research.

In Table 7, review’s primary limitation lies in its descriptive rather than analytical treatment of computational models for protein–protein interaction (PPI) prediction. Although a wide range of approaches—spanning classical machine learning, deep learning and transformer‐based architectures—are mentioned, the manuscript fails to critically analyse their architectural design choices, input representations, preprocessing strategies and evaluation methodologies. As summarised in Table 7, these aspects vary substantially across studies and directly influence model performance and generalisability. Most classical machine learning approaches rely on handcrafted sequence descriptors and shallow classifiers, offering interpretability but limited capacity to model complex interaction patterns. Deep learning models, particularly CNN‐ and RNN‐based approaches, improve representational power but differ significantly in how they encode protein sequences, handle variable sequence lengths and mitigate class imbalance. Transformer‐based and large‐scale pretrained models further advance contextual understanding but introduce new challenges related to computational cost and reproducibility. Despite these critical differences, the manuscript does not systematically evaluate how such design decisions affect PPI prediction outcomes.

**TABLE 7:** Comparative analysis of computational models for protein–protein interaction (PPI) prediction.

Study/model	Model architecture	Input representation	Data preprocessing	Evaluation metrics	Key limitations
Classical ML (SVM, RF, ELM)	Shallow classifiers	Handcrafted sequence descriptors, physicochemical features	Redundancy filtering, feature normalisation	Accuracy, precision	Limited feature expressiveness, poor scalability
CNN‐based PPI models	1D CNN, multilayer CNN	One‐hot encoding, physicochemical vectors, PSSM	Fixed‐length padding, negative sampling	Accuracy, F1‐score, AUC	Limited long‐range dependency modelling
RNN/LSTM models	LSTM, BiLSTM	Sequential amino acid embeddings	Sequence truncation, normalisation	Accuracy, recall	High computational cost, vanishing gradients
Autoencoder‐based models	Stacked/denoising autoencoders	Latent representations from sequence features	Noise injection, dimensionality reduction	Accuracy, MCC	Opaque representations, tuning sensitivity
Hybrid CNN–RNN models	CNN + LSTM/GRU	Local + sequential embeddings	Multistage preprocessing	F1‐score, AUC	Complex training pipelines
Transformer‐based models	Self‐attention, ProteinBERT	Contextual protein embeddings	Large‐scale pretraining, tokenisation	Accuracy, AUC, MCC	High computational and memory cost
Structure‐aware models	CNN + structure features	Sequence + predicted 3D structures	Structure alignment, residue mapping	Precision, recall	Dependence on accurate structure prediction
Proposed MCONet	Multichannel CNN	Sequence‐based numerical encoding	Standardised padding, dropout regularisation	Accuracy, precision, recall, F1, MCC	Requires cross‐dataset validation

Furthermore, the ‘proposed methodology’ section is underdeveloped, providing only a high‐level pseudocode without essential experimental details. Key components such as dataset selection, preprocessing steps, train–test split strategy, baseline comparisons and validation metrics are absent. Without empirical results or controlled experimental design, the proposed method does not contribute substantively beyond conceptual description. In contrast, a meaningful methodology section should clearly specify experimental protocols, datasets, evaluation metrics and comparative baselines to support reproducibility and scientific validity.

## 8 Conclusion

For the study of predicting protein sequences and PPIs, much research has been conducted in the computational biology field, and it is still a wide area to explore for researchers. With exposure to the pandemic, the need for predicting possible protein sequences and the possible interactions, which are made by proteins, has become extremely important. This review is based on various digital and computational techniques for predicting protein–protein sequences and PPIs. Various DL models and neural network models have been implemented to achieve the most accurate technique, which is used for the classification as well as prediction of protein sequences based on their functions and their features. Comparative analysis has been done on various methodologies based on their experimental results, which provide information regarding the most accurate technique for existing data for protein sequences and PPI site prediction. From this study, the authors learnt that the SAE model has the lowest accuracy, and the general regression neural network has the highest accuracy of 99.97%. Hence, from this review, it can be said that the use of the general regression neural network technique is the most accurate procedure for predicting protein sequences and PPIs.

This study presents the MCONet framework as a deep learning‐based approach for protein–protein interaction prediction, emphasising architectural design and representation learning strategies rather than relying solely on isolated accuracy improvements. Although the experimental results demonstrate competitive performance across multiple evaluation metrics, it is important to acknowledge that claims of absolute model superiority cannot be conclusively established in the absence of fully controlled experiments and standardised evaluation protocols.

The comparative analysis highlights trends observed in the literature; however, differences in datasets, preprocessing strategies, class distributions and validation schemes across existing studies limit the fairness of direct numerical comparisons. As a result, reported performance gains should be interpreted as indicative of the model's potential rather than definitive evidence of dominance over existing methods. The lack of universally adopted benchmark datasets and uniform experimental settings remains a significant challenge in protein–protein interaction prediction research.

Despite these limitations, the proposed model demonstrates robustness and consistency under the same experimental conditions applied within this study, suggesting its effectiveness as a viable alternative to existing deep learning‐based PPI prediction frameworks. The transparent reporting of architectural configurations, training protocols and evaluation metrics contributes towards improved reproducibility and more meaningful assessment. Future work will focus on validating MCONet under standardised benchmarking frameworks, including cross‐dataset evaluation and independent test sets, to enable fairer comparison and stronger generalisation claims. Additionally, releasing implementation details and reproducible pipelines will further facilitate objective evaluation by the research community. Overall, this work contributes methodologically to ongoing efforts towards more transparent, reproducible and reliable protein–protein interaction prediction models.

## Author Contributions

**Anindya Nag:** conceptualization, data curation, methodology, visualization, writing – original draft, writing – review and editing. **Riya Sil:** data curation, resources, validation, visualization. **Md. Mehedi Hassan:** resources. **Tamanna Haque Ritu:** resources, supervision, writing – review and editing. **Biva Das:** resources. **Nilanjana Roy:** resources, writing – review and editing. **Anurag Sinha:** conceptualization, data curation, formal analysis. **Dron Guin:** formal analysis, investigation, project administration, visualization. **Nishchal Maurya:** formal analysis, resources.

## Funding

The authors have nothing to report.

## Conflicts of Interest

The authors declare no conflicts of interest.

## Data Availability

The authors have nothing to report.
